# Consensus for the Diagnosis and Treatment of Patients With Spinal Muscular Atrophy (SMA) in Latin America

**DOI:** 10.31083/RN53782

**Published:** 2026-09-23

**Authors:** Andrés E. Nascimento Osorio, Graciela Barros Acevedo, María de los Ángeles Beytía, Edna J. Bobadilla Quesada, Sandra M. Castellar-Leones, Claudia Castiglioni, Marco J. Casartelli Galeano, Guilca Contreras Caicedo, María Fernanda Cordero Molina, Rosa E. Escobar Cedillo, Francisco Espinel, Nicholas Earle, Carlos A. Franco Toñanez, Henry H. Galvez Quiñonez, Juliana Gurgel-Giannetti, Alfonso P. Gutiérrez Mata, María C. Hervias Ruz, Javier E. Linzoain, Mariela A. Lucero, Conrado Medici, Sandra C. Mesa Restrepo, María E. Meza Cano, Soledad Monges, Leticia M. Munive Baez, Lourdes I. Núñez Antelo, Rogelio R. Odales Ibarra, Verónica D. Artiga-Rodríguez, Eva S. Pérez Almengor, Peggy C. Martínez Esteban, Diana Ramírez-Montaño, Rodrigo Moreno-Salgado, Matilde Ruiz García, Verónica Sáez Galaz, Edwin S. Vargas Cañas, Jorge A. Bevilacqua, Edmar Zanoteli, Carol J. Zuniga García, María R. Gueçaimburu, Mario Gutiérrez-Saenz, Jesús A. Armijo Gómez

**Affiliations:** ^1^Unidad de Neuromuscular, Hospital Sant Joan de Déu; Group of Applied Research in Neuromuscular Diseases, Institut de Recerca Sant Joan de Déu, 08950 Barcelona, España; ^2^Center for the Biomedical Research on Rare Diseases (CIBERER), ISCIII, 08036 Barcelona, España; ^3^Centro Hospitalario Pereira Rossell, Instituto de Neurología, 11600 Montevideo, Uruguay; ^4^Teletón Uruguay, 11600 Montevideo, Uruguay; ^5^Sección de Neurología Pediátrica, División de Pediatría, Escuela de Medicina, Pontificia Universidad Católica de Chile, 8331150 Santiago, Chile; ^6^Unidad de Neurología Pediátrica, Servicio de Pediatría, Hospital Dr. Sótero del Río, 8150215 Santiago, Chile; ^7^Departamento de Pediatría, Fundación Hospital de la Misericordia (HOMI), 111411 Bogotá, Colombia; ^8^Hospital Universitario San Ignacio, 110231 Bogotá, Colombia; ^9^Departamento de Medicina Física y Rehabilitación, Facultad de Medicina, Universidad Nacional de Colombia, 111321 Bogotá, Colombia; ^10^Hospital Universitario Nacional de Colombia, 111321 Bogotá, Colombia; ^11^Departamento de Pediatría, Clínica MEDS, 7510155 Santiago, Chile; ^12^Escuela de Medicina, Universidad Finis Terrae, 7501015 Santiago, Chile; ^13^Hospital General Pediátrico Niños de Acosta Ñu, Ministerio de Salud Pública y Bienestar Social, 2160 San Lorenzo, Paraguay; ^14^Servicio de Neuropediatría, Centro Médico Docente La Trinidad, 1080 Caracas, Venezuela; ^15^Neurología Infantil, Clínica Monte Sinaí, 010107 Cuenca, Ecuador; ^16^Clínica Latinoamericana, 010107 Cuenca, Ecuador; ^17^Instituto Nacional de Rehabilitación “Luis Guillermo Ibarra Ibarra” (INR-LGII), 14389 Ciudad de México, Mexico; ^18^Unidad de Neurología Pediátrica, Servicio de Pediatría, Hospital Metropolitano, 170135 Quito, Ecuador; ^19^Departamento de Neurología y Neurocirugía, Clínica Dávila, Santiago, Chile, and Corporación de Investigación de Neurología de Santiago (CINSAN), 8431657 Santiago, Chile; ^20^Departamento de Neuropediatría, Hospital General Pediátrico Niños de Acosta Ñu, 2160 San Lorenzo, Paraguay; ^21^Centro GALO, 01015 Ciudad de Guatemala, Guatemala; ^22^Hospital Regional de Occidente HRO, 09001 Ciudad de Guatemala, Guatemala; ^23^Department of Pediatrics, Faculdade de Medicina da Universidade Federal de Minas Gerais (UFMG), 30130-100 Belo Horizonte, Brazil; ^24^Hospital Nacional de Niños de Costa Rica “Dr. Carlos Sáenz Herrera”, 10101 San José, Costa Rica; ^25^Neurología Infantil, Clínica MEDS, 7510155 Santiago, Chile; ^26^Escuela de Kinesiología, Facultad de Medicina, Universidad de los Andes, 7620001 Santiago, Chile; ^27^Servicio de Neurología Pediátrica, Clínica Universitaria Reina Fabiola, X5000 Córdoba, Argentina; ^28^Hospital Infantil Municipal de la Ciudad de Córdoba, X5000 Córdoba, Argentina; ^29^Servicio de Neurología Infantil, Hospital de Niños Sor María Ludovica, 1900 La Plata, Buenos Aires, Argentina; ^30^Hospital Pablo Tobón Uribe, 050031 Medellín, Colombia; ^31^Hospital CHRISTUS MUGUERZA Alta Especialidad, 64060 Monterrey, Mexico; ^32^Instituto Tecnológico de Monterrey, 64849 Monterrey, Mexico; ^33^Servicio de Neurología, Hospital de Pediatría S.A.M.I.C. “Prof. Dr. Juan P. Garrahan”, 1245 Buenos Aires, Argentina; ^34^Servicio de Neurología, Hospital Médica Sur, 14050 Ciudad de México, Mexico; ^35^Instituto Nacional de Pediatría, 04530 Ciudad de México, Mexico; ^36^VITAE Medicina y Diagnóstico, 10260 Santa Cruz de la Sierra, Bolivia; ^37^Caja Petrolera de Salud, 10260 Santa Cruz de la Sierra, Bolivia; ^38^Hospital Pediátrico Baca Ortiz, 170136 Quito, Ecuador; ^39^Universidad de las Américas (UDLA), 170125 Quito, Ecuador; ^40^Servicio de Neuropediatría, Hospital Nacional de Niños Benjamín Bloom, 1101 San Salvador, El Salvador; ^41^Clínica Neuroplastickids, 1101 San Salvador, El Salvador; ^42^Hospital del Niño, Dr. José Renán Esquivel, 0816-02189 Ciudad de Panamá, Panamá; ^43^Instituto Nacional de Salud del Niño San Borja, 15036 Lima, Perú; ^44^Unidad de Medicina Genómica y Genética, Clínica Imbanaco Grupo Quirón Salud, 760042 Cali, Colombia; ^45^Departamento de Morfología, Facultad de Salud, Universidad del Valle; Hospital Universitario del Valle Evaristo García ESE, 760032 Cali, Colombia; ^46^Fundación Padrino Todos Santos A.C., 23300 Todos Santos, Baja California Sur, Mexico; ^47^Servicio de Neuropsiquiatría Infantil, Hospital Clínico San Borja Arriarán, 8320000 Santiago, Chile; ^48^Clínica Universidad de los Andes, 7620001 Santiago, Chile; ^49^Clínica de Enfermedades Neuromusculares, Instituto Nacional de Neurología y Neurocirugía “Manuel Velasco Suárez”, 14269 Ciudad de México, Mexico; ^50^Centro de Neurociencias, Hospital Ángeles del Pedregal, 01900 Ciudad de México, Mexico; ^51^Departamento de Neurología y Neurocirugía, Hospital Clínico Universidad de Chile & Departamento de Anatomía y Medicina Legal, Facultad de Medicina, Universidad de Chile, 8380453 Santiago, Chile; ^52^Department of Neurology, Faculdade de Medicina, Universidade de São Paulo (FMUSP), 01246-903 São Paulo, Brazil; ^53^Hospital Mario Catarino Rivas, Servicio de Neurología Pediátrica, 21104 San Pedro Sula, Cortés, Honduras; ^54^Hospital del Valle, 21102 San Pedro Sula, Cortés, Honduras; ^55^Equipo de Enfermedades Raras, CRENADECER, BPS, 11200 Montevideo, Uruguay; ^56^Neurología, Hospital Clínica Bíblica, 10101 San José de Costa Rica, Costa Rica; ^57^Caja Costarricense del Seguro Social, 10104 San José de Costa Rica, Costa Rica; ^58^Hospital General con Especialidades Juan María Salvatierra, 23000 La Paz, Mexico; ^59^Neurología Pediátrica & Patología Neuromuscular, 23000 La Paz, Mexico

**Keywords:** spinal muscular atrophies of childhood, spinal muscular atrophy, survival motor neuron 1 gene (*SMN1*), survival motor neuron 2 gene (*SMN2*), nusinersen, risdiplam, genetic therapy, neonatal screening, Latin America, consensus, atrofia muscular espinal, atrofias musculares espinales infantiles, gen de supervivencia de la neurona motora 1 (*SMN1*), gen de supervivencia de la neurona motora 2 (*SMN2*), nusinersen, risdiplam, terapia génica, pesquisa neonatal, América Latina, consenso

## Abstract

**Background:**

5q spinal muscular atrophy (5q SMA) is an autosomal recessive neuromuscular disorder caused by mutations in the survival motor neuron 1 gene (*SMN1*), leading to deficiency of SMN protein and a variable clinical spectrum influenced by survival motor neuron 2 gene (*SMN2*) copy number. The advent of disease-modifying therapies (DMTs) has transformed its clinical course, requiring timely diagnosis, multidisciplinary care, and longitudinal response assessment. In Latin America (LATAM), significant gaps and heterogeneity persist in access to genetic diagnosis, newborn screening, DMTs, and specialized follow-up. To develop a Latin American consensus providing recommendations for diagnosis, comprehensive management (respiratory, swallowing/nutrition, rehabilitation, and DMTs), and evaluation of response to DMTs in patients with SMA, incorporating real-world access considerations.

**Methods:**

A regional expert panel conducted a formal consensus process (2025–2026) through iterative questionnaires and structured discussions. Agreement was defined as ≥70% concordance. Additionally, a regional survey assessed access to diagnostic and therapeutic resources.

**Results:**

Forty professionals from 15 countries participated. While 90% reported access to genetic testing (MLPA), only 35% indicated availability within the public healthcare system; private access and pharmaceutical industry support were also reported as pathways to testing. No participating country reported nationwide implementation of newborn screening for SMA; 22.5% of respondents reported pilot programs or future implementation proposals. Access to at least one DMT was reported by 92.5% of respondents; however, delays between diagnosis and treatment initiation were frequent and remain an important challenge across the region. Consensus recommendations were established for diagnosis, functionally stratified multidisciplinary care, and response assessment based on motor and non-motor domains using validated tools and individualized follow-up.

**Conclusions:**

This consensus provides a practical and adaptable framework to optimize SMA care in LATAM, supporting longitudinal, patient-centered clinical decision-making.

## Abstract (es)

**Antecedentes:**

La atrofia muscular espinal ligada al cromosoma 5q (AME 5q) es una enfermedad neuromuscular genética autosómica recesiva causada por mutaciones en el gen *SMN1*, que generan deficiencia de la proteína de supervivencia de la neurona motora (SMN) y un espectro clínico variable influido por el número de copias de *SMN2*. La introducción de terapias modificadoras de la enfermedad (TME) ha transformado su evolución, requiriendo diagnóstico oportuno, abordaje multidisciplinario y evaluación longitudinal de la respuesta. En América Latina (LATAM), persisten brechas relevantes y heterogeneidad en el acceso a diagnóstico genético, pesquisa neonatal, TME y seguimiento especializado. Desarrollar un consenso latinoamericano con recomendaciones para el diagnóstico, tratamiento integral (respiratorio, deglución/nutrición, rehabilitación y TME) y evaluación de la respuesta a TME en pacientes con AME, incorporando información contextual sobre acceso en la región.

**Métodos:**

Se conformó un panel regional de expertos que trabajó entre 2025 y 2026 mediante un proceso de consenso formal con rondas sucesivas de cuestionarios y discusión estructurada. Se definió acuerdo con ≥70% de concordancia. Adicionalmente, se realizó una encuesta regional para evaluar el acceso a recursos diagnósticos y terapéuticos.

**Resultados:**

Participaron 40 profesionales de 15 países. Mientras que el 90% de los encuestados informó tener acceso a pruebas genéticas (MLPA), solo el 35% indicó que estas estaban disponibles dentro del sistema público de salud; también se señalaron el acceso privado y el apoyo de la industria farmacéutica como vías para realizar las pruebas. Ningún país participante informó la implementación a nivel nacional del cribado neonatal para AME; el 22,5% de los encuestados informó la existencia de programas piloto o propuestas de implementación futura. El acceso a al menos una terapia modificadora de la enfermedad (TME) fue informado por el 92,5% de los encuestados; sin embargo, los retrasos entre el diagnóstico y el inicio del tratamiento fueron frecuentes y continúan siendo un desafío importante en la región. Se consensuaron recomendaciones diagnósticas, manejo multidisciplinario estratificado por nivel funcional y criterios de evaluación de respuesta basados en dominios motores y no motores con herramientas validadas y seguimiento individualizado.

**Conclusiones:**

Este consenso proporciona un marco práctico y adaptable para optimizar la atención de la AME en LATAM, promoviendo decisiones clínicas longitudinales y centradas en el paciente.

## 1. Introduction

Spinal muscular atrophy (SMA) comprises a heterogeneous group of rare hereditary neuromuscular disorders characterized by the progressive degeneration of anterior horn motor neurons in the spinal cord and bulbar motor nuclei in the brainstem, leading to variable patterns of muscle weakness and atrophy, frequently associated with respiratory and bulbar involvement, and high mortality in the most severe early-onset forms [1,2].

From a genetic perspective, SMA is broadly classified into two major categories: chromosome 5q-linked SMA (5q SMA), which accounts for approximately 90–95% of cases and has an estimated incidence of 1 in 5000–10,000 live births [3,4].

In most cases, 5q SMA is caused by homozygous deletions of exon 7 in the *SMN1* gene and, less frequently, by point mutations, resulting in a critical deficiency of survival motor neuron (SMN) protein [5,6]. The paralogous *SMN2* gene acts as the primary phenotypic modifier by producing limited amounts of functional SMN protein; accordingly, a higher *SMN2* copy number is generally associated with milder clinical phenotypes, creating a continuous disease spectrum ranging from prenatal-onset forms to adult manifestations [3,6,7].

This Latin American consensus specifically focuses on 5q SMA, comprehensively addressing diagnostic, therapeutic, and multidisciplinary management aspects within the current clinical landscape.

Over the past decade, the therapeutic landscape of SMA has undergone substantial transformation with the introduction of disease-modifying therapies (DMTs) aimed at restoring SMN protein production. Pivotal clinical trials have demonstrated that treatments such as nusinersen, risdiplam, and gene therapy with onasemnogene abeparvovec significantly improve survival, motor function, and developmental milestones, particularly when administered during presymptomatic stages of the disease [8,9,10,11,12,13,14,15,16,17,18,19].

These advances have shifted SMA from a rapidly progressive and frequently fatal disease into a chronic condition requiring long-term follow-up [1,8,20].

However, the care of individuals with SMA extends beyond DMTs alone and requires a comprehensive management approach [1,21,22]. International standards of care emphasize the importance of multidisciplinary management that includes respiratory care, swallowing and nutritional assessment, motor rehabilitation, orthopedic surveillance, and systematic monitoring of motor and respiratory function [1,21,22].

The integration of these interventions is essential to optimize clinical outcomes, prevent complications, and improve patient quality of life [21,22,23] (Fig. 1).

**Fig. 1.**
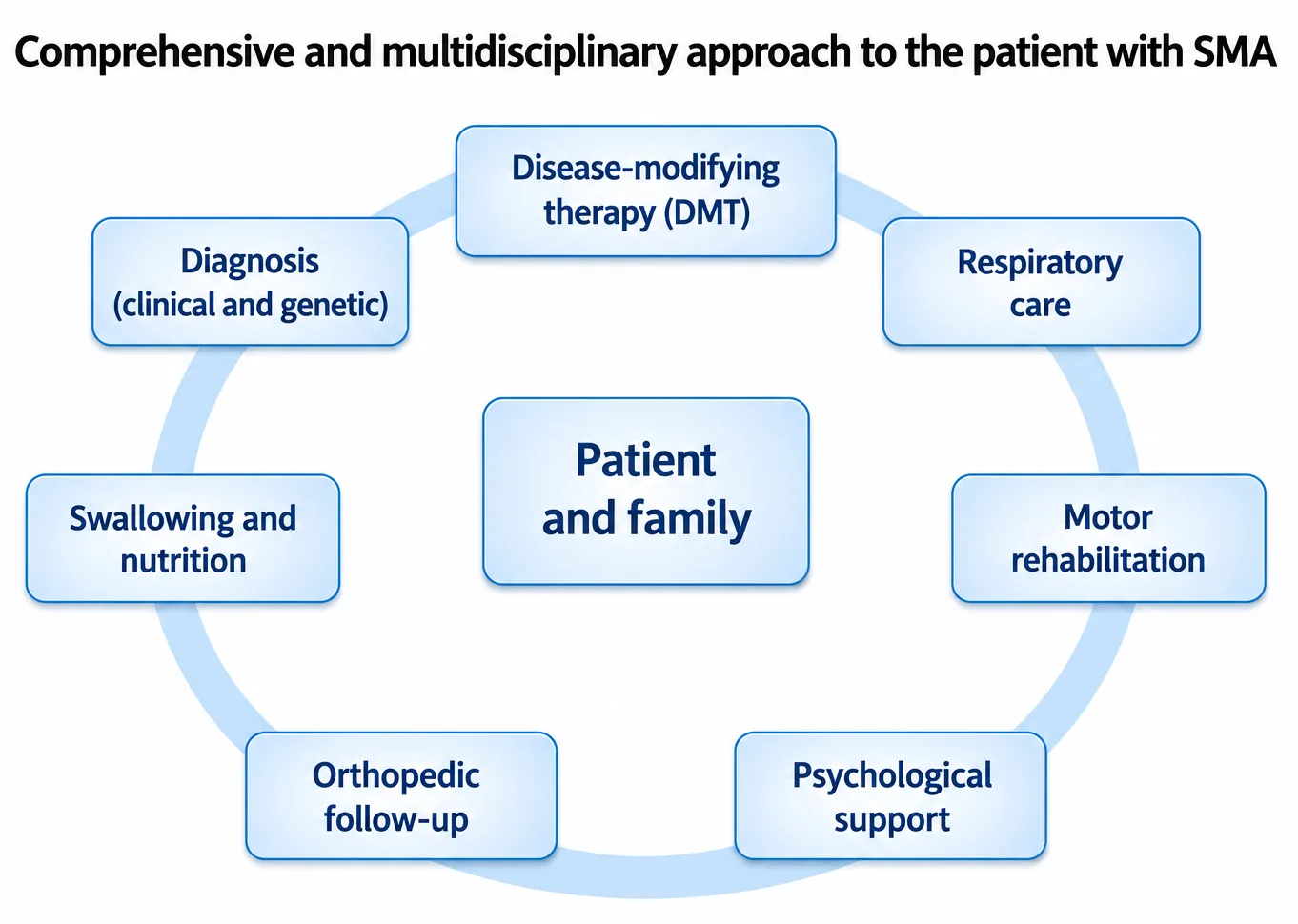
**Components of comprehensive multidisciplinary management in patients with spinal muscular atrophy**. Ref: Conceptual framework of comprehensive management for spinal muscular atrophy, including early diagnosis, disease-modifying therapy (DMT), and continuous multidisciplinary care (respiratory, nutritional, swallowing, rehabilitation, orthopedic follow-up, and functional monitoring), all of which are necessary to optimize clinical outcomes and quality of life. SMA, spinal muscular atrophy.

In Latin America (LATAM), implementation of international SMA care standards faces significant challenges due to heterogeneous healthcare systems, regulatory differences among countries, and variable availability of confirmatory genetic diagnosis. Additional barriers include geographic limitations, restricted access to referral centers and newborn screening programs, as well as inequities in access to DMTs and multidisciplinary teams with adequate expertise and resources for comprehensive patient care [24,25].

These disparities contribute to diagnostic delays, variability in therapeutic decision-making, and difficulties in the longitudinal follow-up of SMA patients across the region [24,26].

Consequently, there is a critical need to develop regional recommendations that remain aligned with scientific evidence and international consensus standards—primarily established in high-income settings—while accounting for the specific realities of the Latin American context and serving as a practical tool for clinical decision-making [1,27,28].

The primary objective of this Latin American consensus is to provide recommendations for diagnosis, comprehensive treatment (including respiratory, swallowing, motor rehabilitation, and DMT-related aspects), monitoring, and treatment response assessment in patients with SMA across LATAM. A secondary objective is to incorporate contextual information regarding access to care and DMTs throughout the region.

## 2. Methods

A regional panel of experts in neuromuscular diseases with experience in the diagnosis, treatment, and longitudinal care of patients with SMA was convened from Latin American countries. Experts were identified and invited by the two consensus coordinators (AENO and JAAG) based on their recognized clinical experience in SMA and active involvement in the care and follow-up of patients with the disease, while also seeking broad geographic representation across Latin America. The panel included clinicians from relevant specialties, including genetics, all actively involved in the care and management of patients with SMA. The coordinators led the project from its conception through completion. A structured consensus-development process was conducted during 2025 and 2026, consisting of an initial virtual meeting to define the scope and objectives, three successive rounds of questionnaires, and two subsequent virtual meetings for discussion and final refinement of the recommendations. Participants received relevant scientific literature together with the questionnaires to support evidence-informed responses beyond individual clinical experience [29]. To develop the consensus, a structured methodology based on iterative questionnaire rounds and discussion of agreements, disagreements, clinical scenarios, and management situations was employed [29]. A priori, agreement was defined as ≥70% homogeneous responses among participants, uncertainty as 40–69%, and lack of agreement as <40%. Statements not reaching agreement during the initial round were reviewed for clarity and applicability, reformulated when necessary, supplemented with additional scientific evidence, and reassessed in subsequent rounds. Statements that remained without consensus after the iterative rounds were discussed by the full panel; when consensus could not ultimately be achieved, they were not presented as formal consensus recommendations [29]. Statement development was stratified into recommendations concerning: (A) diagnosis; (B) treatment (respiratory management, swallowing, rehabilitation, and disease-modifying therapies [DMTs]); and (C) response to DMTs in SMA patients. In this document, the term DMT includes nusinersen, risdiplam, and gene therapy with onasemnogene abeparvovec. The principal statistical methods used included measures of central tendency and dispersion: mean, median, mode, maximum, minimum, and standard deviation.

Once recommendations were defined, manuscript drafting proceeded and was subsequently circulated among all participants for final approval and dissemination.

Secondarily, with the objective of complementing consensus recommendations with contextual information regarding real-world access to SMA diagnosis and treatment in LATAM, a specific survey was developed and directed to consensus participants. The survey was designed by the coordinating group and subsequently validated by the working group to evaluate aspects related to clinical practice, availability of diagnostic and therapeutic resources, and the principal barriers to access across different healthcare settings. The questionnaire was originally developed in Spanish, and no translations into other languages were performed. Prior to definitive implementation, the instrument underwent pilot validation in two successive phases. Each pilot phase included four physicians with SMA expertise, who evaluated question clarity, relevance of explored domains, and feasibility of implementation [30,31]. Based on the feedback received, adjustments were made to the wording and organization of items until the final questionnaire version was obtained. The final survey was structured to collect information in the following domains: (a) sociodemographic and professional characteristics of respondents; (b) availability of tools for SMA diagnosis; and (c) availability of and access conditions to DMTs for SMA. Survey responses were analyzed at the individual respondent level and were not aggregated to assign country-level classifications; therefore, differing responses from participants within the same country were retained as reported. Final versions of the questionnaires used are included as **Supplementary Material 1**. Survey results should be interpreted as descriptive and not necessarily representative of the entire Latin American region.

## 3. Results

During the September–October 2025 period, a total of 40 healthcare professionals from 15 Latin American countries responded to the survey: Argentina, Bolivia, Brazil, Chile, Colombia, Costa Rica, Ecuador, El Salvador, Honduras, Mexico, Panama, Paraguay, Peru, Uruguay, and Venezuela (Table 1, Fig. 2). Most respondents practiced in urban areas with populations exceeding one million inhabitants (85.0%), while 12.5% practiced in large cities (100,000–1 million inhabitants) and 2.5% in smaller cities. Regarding patient population, 67.5% primarily treated pediatric patients, 22.5% treated both children and adults, and 10.0% exclusively treated adults.

**Table 1.** **Characteristics of respondents and access to SMA diagnosis and treatment**.

Domain	Variable	n	%
Respondent characteristics	Participating countries	15	—
Respondent characteristics	Participating professionals	40	100
Respondent characteristics	Practice in urban area (>1 million inhabitants)	34	85.0
Respondent characteristics	Primarily pediatric care	27	67.5
Diagnosis	Access to MLPA	36	90.0
Diagnosis	MLPA result ≤4 weeks	32	80.0
Diagnosis	Newborn screening unavailable	31	77.5
Comprehensive care	Multidisciplinary units available	31	77.5
Comprehensive care	≥80% access to multidisciplinary management	23	57.5
DMTs	Access to ≥1 DMT	37	92.5
DMTs	Public and private access	26	65.0
DMTs	Treatment initiation within 1–6 months	17	42.5

DMTs, disease-modifying therapies; MLPA, multiplex ligation-dependent probe amplification.

**Fig. 2.**
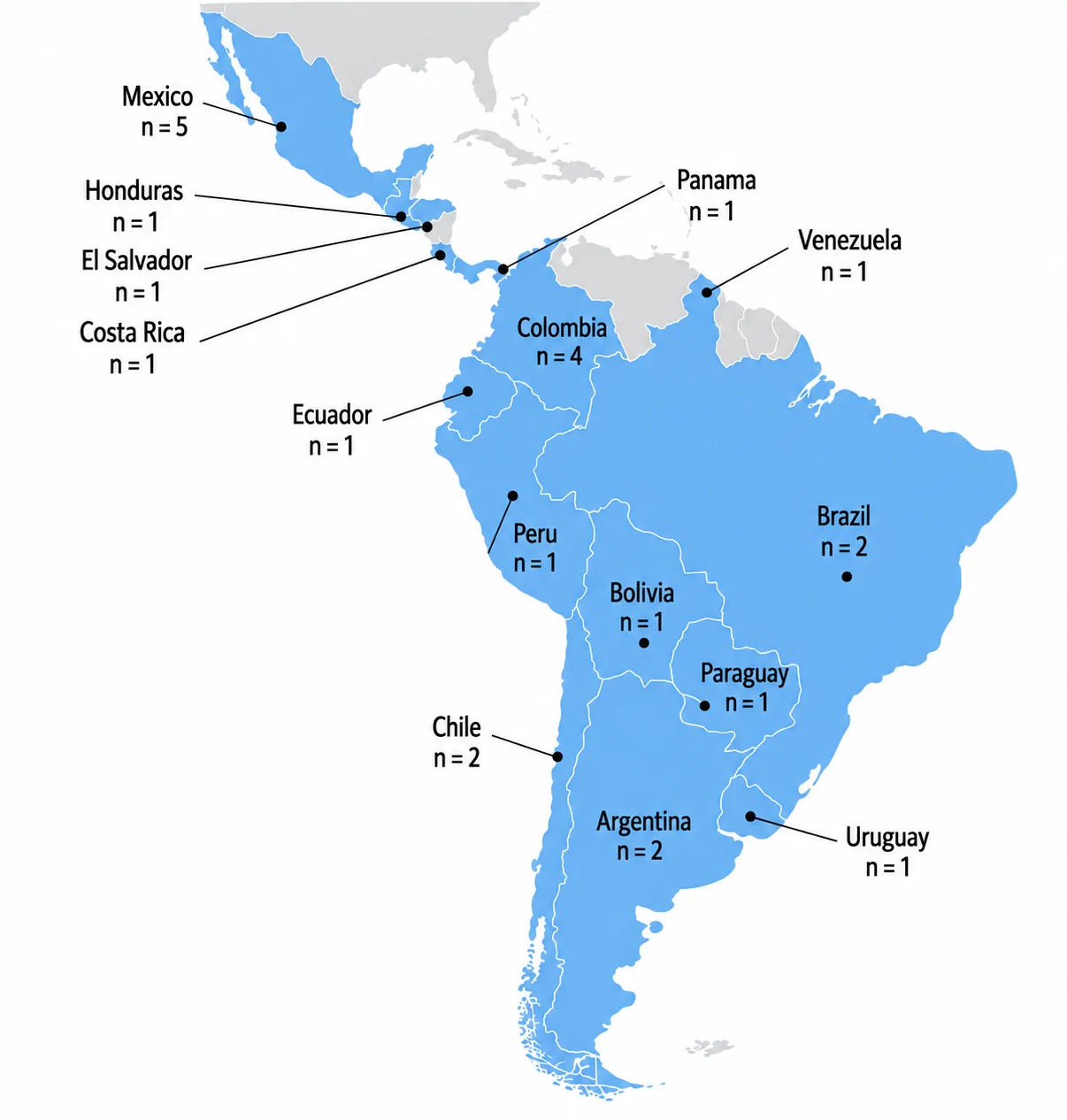
**Geographic distribution of participating experts across Latin America**. Ref: Geographic distribution of participating experts across Latin America. The map shows the 15 Latin American countries represented in the consensus process and the number (n) of participating experts from each country. A total of 40 experts participated in the consensus.

Regarding the availability of diagnostic tools, 90.0% of respondents reported having access to MLPA testing for SMA diagnosis. The estimated turnaround time for test results was ≤4 weeks in 80.0% of cases, while 15.0% reported delays of 4–8 weeks and the remaining group reported delays exceeding 8 weeks. Concerning access pathways to genetic testing, 35.0% indicated that testing was available within the public healthcare system, 35.0% reported private access, and 30.0% required support from the pharmaceutical industry for test completion.

With respect to newborn screening for SMA, 77.5% of participants reported that it is not currently implemented in their country, while 22.5% reported its existence as either a pilot study or future proposal.

Regarding comprehensive care, 77.5% of respondents reported the existence of multidisciplinary units with experience in SMA patient management within their country. However, only 12.5% estimated that 100% of patients had access to multidisciplinary care, while 45.0% reported approximately 80% access, 20.0% reported 50% access, and 22.5% estimated that fewer than 20% of patients had such access (Table 1 and Fig. 3). The availability of home mechanical ventilation programs, whether invasive or non-invasive, was reported by 57.5% of participants.

**Fig. 3.**
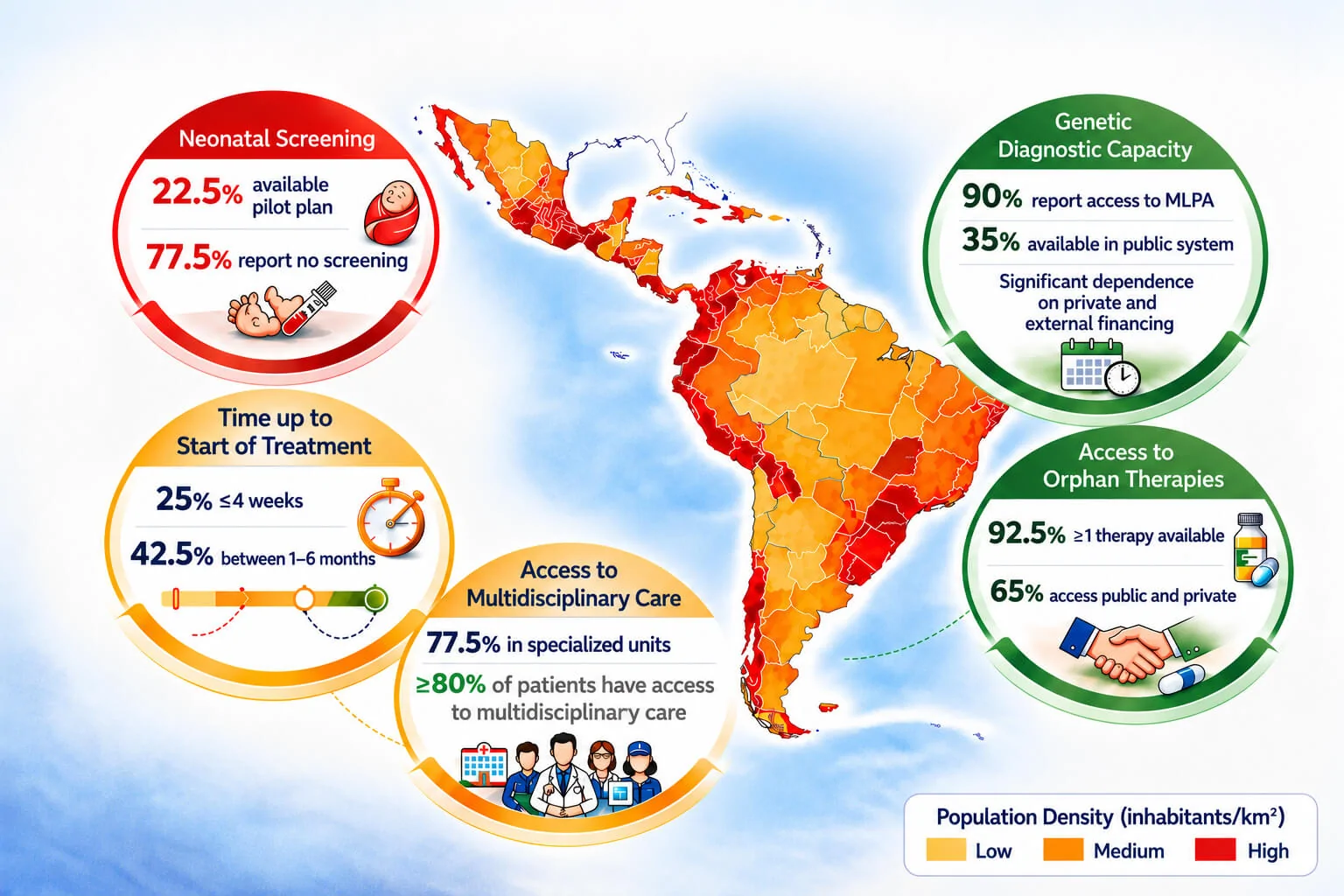
**Overview of access to spinal muscular atrophy care in Latin America: key indicators**. Ref: Conceptual map of heterogeneity in access to diagnosis and treatment of spinal muscular atrophy (SMA) in Latin America. Representation based on aggregated results from a regional survey (n = 40 professionals, 15 countries). Traffic light color coding: green = high availability; yellow = intermediate availability; red = low availability.

Regarding access to DMTs, 92.5% of respondents reported availability of at least one approved therapy for SMA, while 7.5% indicated no access to disease-modifying therapies. Access to these treatments was predominantly described as both public and private in 65.0% of cases, exclusively public in 27.5%, and exclusively private in 5.0%.

The estimated time from diagnosis to DMT initiation was ≤4 weeks in 25.0% of cases, while 42.5% reported delays ranging from 1 to 6 months, and 20.0% reported delays exceeding 6 months.

Finally, 55.0% of respondents reported the existence of a health committee responsible for determining patient eligibility for treatment.

## 4. Recommendations for the Diagnosis and Treatment of Patients With Spinal Muscular Atrophy

### 4.1 Recommendations for the Diagnostic Process of SMA

The diagnosis of 5q SMA should be based on the genetic confirmation of biallelic pathogenic variants in the *SMN1* gene, with homozygous deletion of exon 7 representing the most frequent and clinically relevant alteration, present in approximately 95% of patients regardless of phenotypic severity [1,32]. In patients with a typical phenotype, particularly in early-onset presentations, diagnosis can usually be confirmed through molecular studies without requiring additional evaluations. In this context, the panel recommends MLPA or quantitative PCR as the initial diagnostic test for detecting SMN1 exon 7 deletion, reserving SMN1 sequencing for cases with heterozygous deletion or atypical genetic findings in order to identify point mutations not detectable by gene dosage-based methods [1,32,33] (Table 2).

**Table 2.** **Recommendations for the diagnostic process of SMA**.

**For the diagnosis and early detection of SMA, the following are recommended:**
(a) Confirm the diagnosis through demonstration of biallelic pathogenic variants in the *SMN1* gene.
(b) Include detection of *SMN1* exon 7 deletion using MLPA or quantitative PCR techniques as the initial diagnostic confirmation study.
(c) Determine *SMN2* copy number as part of the diagnostic process.
(d) Perform *SMN1* gene sequencing in cases where a single deletion is identified or atypical genetic findings are obtained, with the objective of excluding point mutations.
(e) Consider other neuromuscular diseases and expand complementary studies according to the characteristics of each case in patients with atypical clinical presentation or without genetic confirmation.
(f) Implement newborn screening for SMA in order to promote early disease detection.

Additionally, determination of SMN2 copy number is recommended as an integral part of the diagnostic algorithm. Although it does not constitute a diagnostic criterion per se, it is an important prognostic marker, given its inverse correlation with clinical severity and its contribution to anticipating disease course, including during presymptomatic stages [1,7,33].

Finally, the panel recommends advancing the implementation of SMA newborn screening programs. This strategy is supported by management algorithms for infants diagnosed through newborn screening, regional pilot experiences such as those reported in Brazil [20,28,34], as well as consolidated population-based programs such as the one implemented in Germany, which have demonstrated that presymptomatic diagnosis and treatment are associated with a significantly greater likelihood of achieving major motor milestones, including independent ambulation, compared with symptomatically diagnosed cohorts [15,35].

Presymptomatic detection facilitates the early initiation of disease-modifying therapies with clinically meaningful impact, in alignment with international standards of care [1,15,35] (Table 2).

### 4.2 Recommendations for Multidisciplinary Treatment

At this stage, patients were stratified into three groups to facilitate recommendations: those who had not achieved sitting, those who had achieved sitting, and those who were ambulatory.

#### 4.2.1 Patients With SMA Who Have Not Achieved Sitting

In patients with SMA who have not achieved sitting, the panel recommends interpreting treatment response broadly, considering both improvement and clinical stability as relevant outcomes (Table 3.1). This approach is based on the progressive natural history of this subgroup, in which the absence of deterioration constitutes a clinically meaningful therapeutic effect [1,27,36].

**Table 3.1.** **Recommendations for patients with SMA who have not achieved sitting**.

**Recommendations for respiratory management**	**Recommendations for swallowing management**	**Recommendations for rehabilitation management**
(a) Evaluate respiratory function through physical examination, including respiratory rate and amplitude, cough effectiveness, and other functional aspects.	(a) Evaluate swallowing function through physical examination, including suction, chewing efficiency, presence of clinical signs of aspiration, and other functional aspects.	(a) Clinically evaluate postural control, presence and degree of scoliosis, and range of joint motion in order to guide rehabilitation strategy.
(b) Perform respiratory function assessment as a screening method using oximetry and transcutaneous capnography.	(b) Indicate instrumental swallowing assessment through videofluoroscopic swallow study in the presence of clinical signs of swallowing disorders.	(b) Assess for the presence of orthopedic deformities, including foot deformities, Achilles tendon contracture, kyphosis, and hip dislocation.
(c) Indicate a sleep study (polysomnography) in the presence of clinical suspicion of hypoventilation.	(c) Evaluate for the possible presence of gastroesophageal reflux.	(c) Assess the magnitude of muscle weakness using validated functional scales such as CHOP INTEND.
(d) Implement respiratory hygiene and support strategies, including oronasal suctioning, respiratory rehabilitation/physiotherapy, and assisted cough techniques, among others.		(d) Evaluate motor development using standardized scales such as HINE.
(e) Indicate non-invasive ventilation in the presence of clinically evident hypoventilation confirmed by complementary studies.		(e) Indicate daily use of seating systems, postural supports, and positioning devices in patients with postural disorders in order to promote alignment and postural correction.
		(f) Indicate daily use of limb orthoses in patients with contractures in order to promote stretching, function, and maintenance of joint range of motion.
		(g) Recommend that orthoses, when indicated, be used for as long as the patient can tolerate in order to optimize intervention effectiveness and functionality.
		(h) Indicate stretching exercises once or twice daily to improve joint range of motion.
		(i) Recommend that the minimum frequency of stretching be at least 5 to 7 times per week to achieve an adequate therapeutic effect.
		(j) Recommend that, in patients using orthoses, the minimum frequency of use should be at least 5 times per week to ensure intervention effectiveness.
**Recommendations for disease-modifying therapies**		
(a) Nusinersen has demonstrated efficacy and safety for the treatment of the disease. Its use should be evaluated by the treating team with the objective of modifying disease course.		
(b) Risdiplam has demonstrated efficacy and safety for the treatment of the disease. Its use should be evaluated by the treating team with the objective of modifying disease course.		
(c) Onasemnogene abeparvovec has demonstrated efficacy and safety for the treatment of the disease in patients younger than 2 years of age. Its use should be evaluated by the treating team with the objective of modifying disease course.		

The consensus statements emphasize the need to assess response using objective functional scales adapted for non-sitter patients, capable of detecting subtle and reproducible changes. In this context, evaluation should not be limited to the acquisition of major motor milestones, but rather should focus on overall functional evolution and small yet sustained clinically relevant changes [1,37].

Additionally, incorporation of respiratory and bulbar parameters as valid response criteria is recommended, given that respiratory stability or improvement, reduction of infectious complications, and preservation of oral feeding constitute indicators of high clinical value, in accordance with Table 3.1 [1,22].

Regarding assessment frequency, consensus established that evaluation intervals should be flexible and adjusted to individual clinical circumstances, balancing follow-up needs with implementation feasibility in real-world regional care settings [1,22,27].

Finally, consensus was reached to maintain DMTs even in the absence of objectively measurable short-term improvement, considering that sustained clinical stability across defined functional, respiratory, and bulbar domains constitutes a valid argument for treatment continuation [1,22,27] (Table 3.1).

#### 4.2.2 Patients With SMA Who Have Achieved Sitting

In patients with SMA who have achieved sitting, the panel reached consensus that evaluation of response to DMTs should adopt a comprehensive functional approach (Table 3.2). This subgroup presents an intermediate clinical profile, with the possibility of prolonged stability and, in some cases, partial functional improvement [1,2,22].

**Table 3.2.** **Recommendations for patients with SMA who have achieved sitting**.

**Recommendations for respiratory management**	**Recommendations for swallowing management**	**Recommendations for rehabilitation management**
(a) Evaluate respiratory function through physical examination, including respiratory rate and amplitude, cough effectiveness, and other functional aspects.	(a) Evaluate swallowing function through physical examination, including suction, chewing efficiency, presence of clinical signs of aspiration, and other functional aspects.	(a) Clinically evaluate postural control, presence and degree of scoliosis, and range of joint motion in order to design or adapt an appropriate rehabilitation program.
(b) Perform respiratory function assessment as a screening method using oximetry.	(b) Indicate instrumental swallowing assessment through videofluoroscopic swallow study in the presence of clinical signs of swallowing disorders.	(b) Assess for the presence of orthopedic deformities, including foot deformities and hip dislocation, which may require specific treatment.
(c) Indicate respiratory function assessment through spirometry with measurement of peak cough flow (PCF), whenever patient age and degree of cooperation allow.		(c) Use the CHOP INTEND scale to assess the degree of weakness in patients ≤24 months of age and/or with significant weakness.
(d) Indicate a sleep study (polysomnography) in the presence of clinical suspicion of hypoventilation.		(d) Evaluate the presence and degree of muscle weakness using validated functional scales such as EK2.
(e) Evaluate for the possible presence of gastroesophageal reflux.		(e) Apply additional functional scales during follow-up, such as HFMSE, RULM, and MFM, with the objective of evaluating changes in motor function.
(f) Indicate non-invasive ventilation in the presence of clinically evident hypoventilation confirmed by complementary studies.		(f) Indicate daily use of seating systems, postural supports, and positioning devices in patients with postural disorders in order to promote alignment and postural correction.
		(g) Indicate daily use of limb orthoses in patients with contractures in order to promote stretching, function, and maintenance of joint range of motion.
		(h) Recommend that orthoses, when indicated, be used for as long as the patient can tolerate in order to optimize intervention effectiveness.
		(i) Recommend that the minimum frequency of stretching be at least 5 to 7 times per week to improve joint range of motion.
		(j) Recommend that, in patients using orthoses, the minimum frequency of use should be at least 5 times per week to ensure intervention effectiveness.
**Recommendations for disease-modifying therapies**		
(a) Nusinersen has demonstrated efficacy and safety for the treatment of the disease. Its use should be evaluated by the treating team with the objective of modifying disease course.		
(b) Risdiplam has demonstrated efficacy and safety for the treatment of the disease. Its use should be evaluated by the treating team with the objective of modifying disease course.		
(c) Onasemnogene abeparvovec has demonstrated efficacy and safety for the treatment of the disease in patients younger than 2 years of age. Its use should be evaluated by the treating team with the objective of modifying disease course.		

The consensus statements highlight the utility of objective motor scales appropriate for sitting patients, which allow quantification of clinically meaningful changes in axial, proximal, and upper limb function. In this context, response should not be interpreted solely as the acquisition of new motor milestones, but also as preservation of previously attained abilities, a particularly relevant consideration in a progressive disease [1,37,38].

Additionally, incorporation of respiratory parameters is recommended, considering functional stability, absence of progression in ventilatory support requirements, and reduction of acute respiratory events as clinically meaningful outcomes, in line with the evaluated domains [1,22].

The panel also recommends incorporating bulbar and nutritional criteria, such as stability of oral feeding and reduction of associated complications, given their relevance to quality of life and overall disease progression in this subgroup [1,22].

Regarding periodicity, a flexible evaluation frequency adjusted to clinical status and resource availability is recommended, aiming for realistic implementation without compromising detection of clinically meaningful changes [1,22].

Finally, continuation of DMTs is considered appropriate even in the absence of objectively measurable short-term motor improvement, provided sustained clinical stability exists across the agreed-upon domains [1,22,27] (Table 3.2).

#### 4.2.3 Ambulatory Patients With SMA

In patients with SMA who have achieved ambulation, the panel reached consensus that evaluation of response to DMTs should prioritize preservation of walking ability and ambulatory motor function, as their loss constitutes a critical clinical milestone with functional and prognostic impact [1,2,22,27] (Table 3.3).

**Table 3.3.** **Recommendations for ambulatory patients with SMA**.

**Recommendations for respiratory management**	**Recommendations for swallowing management**	**Recommendations for rehabilitation management**
(a) Undergo physical examination for respiratory function assessment, including respiratory rate, amplitude, cough effectiveness, and other functional aspects.	(a) Undergo physical examination for swallowing function assessment, including suction, chewing efficiency, presence of clinical signs of aspiration, and other functional aspects.	(a) Clinically evaluate postural control, presence and degree of scoliosis, and range of joint motion in order to design or adapt a rehabilitation program according to individual patient needs.
(b) Undergo spirometry evaluation whenever age and degree of cooperation allow.	(b) Undergo videofluoroscopic swallow study in the presence of clinical signs of swallowing disorders.	(b) Use functional scales such as HFMSE, RULM, and Timed Function Tests (TFTs) during follow-up to evaluate changes in functional status.
(c) Undergo sleep study (polysomnography) in the presence of clinical suspicion of hypoventilation.	(c) Undergo periodic nutritional evaluation by specialists to assess weight, height, caloric intake, and identify signs of malnutrition.	(c) Identify the presence of falls during ambulation for specific incorporation into the rehabilitation plan.
		(d) Indicate daily use of limb orthoses in the presence of contractures, with the objective of promoting stretching, function, and maintenance of joint range of motion.
**Recommendations for disease-modifying therapies**		
(a) Nusinersen has demonstrated efficacy and safety for the treatment of the disease. Its use should be evaluated by the treating team with the objective of modifying disease course.		
(b) Risdiplam has demonstrated efficacy and safety for the treatment of the disease. Its use should be evaluated by the treating team with the objective of modifying disease course.		
(c) Onasemnogene abeparvovec has demonstrated efficacy and safety for the treatment of the disease in patients younger than 2 years of age. Its use should be evaluated by the treating team with the objective of modifying disease course.		
**Recommendations for disease-modifying therapies in patients older than 18 years**		
(a) Nusinersen has demonstrated efficacy and safety for the treatment of the disease.		
(b) Use of nusinersen should be evaluated by the treating team with the objective of modifying disease course.		
(c) Risdiplam has demonstrated efficacy for the treatment of the disease.		
(d) Use of risdiplam should be evaluated by the treating team with the objective of modifying disease course		

In this subgroup, therapeutic response is more commonly expressed as slowing or stabilization of deterioration rather than as evident motor gain; therefore, the use of sensitive scales and functional tests for gait, endurance, and motor performance is essential to detect clinically meaningful changes [1,22,39].

The panel also emphasizes the importance of longitudinal follow-up during adolescence and early adulthood, periods in which functional decline may be observed even in clinically stable patients [1,22].

Additionally, systematic respiratory function assessment is recommended, considering that respiratory involvement may develop insidiously and coexist with apparently preserved ambulation [1,22].

In adults with SMA (≥18 years), a multidimensional approach focused on preserving function and preventing loss of abilities is recommended, recognizing that treatment response may primarily manifest as sustained clinical stability. In the absence of improvement or stabilization, continuation of disease-modifying treatment should be determined based on a comprehensive clinical analysis that considers individualized patient goals impacting activities of daily living, including respiratory and bulbar parameters as well as patient-perceived disease burden [1,22,26,39].

In this context, sustained functional stability constitutes a clinically meaningful outcome, particularly when available assessment tools may lack sufficient sensitivity to detect significant changes [22,27,39,40] (Tables 3.3 and 3.4).

**Table 3.4.** **Cross-Cutting recommendations common to all functional states**.

**Recommendations**	
**Multidisciplinary follow-up**	• Conduct comprehensive clinical follow-up through a multidisciplinary team with expertise in SMA.
	• Adjust evaluation frequency according to patient age and functional status, based on the treating team’s clinical judgment.
	• Ensure periodic specialist evaluation according to organ system involvement.
**Minimum follow-up frequency**	• Perform periodic evaluations at a minimum frequency of every 3 to 6 months.
**Caregiver training**	• Provide training to primary caregivers for respiratory, nutritional, and rehabilitation management as appropriate.

### 4.3 Recommendations on Treatment Response

Evaluation of response to DMTs in SMA requires a comprehensive approach that accounts for patients’ clinical, functional, and disease-course heterogeneity. The panel recommends that treatment response should not be interpreted in an isolated or cross-sectional manner, but rather within a longitudinal, individualized framework that incorporates multiple disease domains or dimensions, in accordance with evidence from clinical trials, real-world data, and international consensus statements [1,8,22,26,27] (Table 4).

**Table 4.** **Recommendations on response to disease-modifying therapy (DMT)**.

**I-Determinants of Response to DMTs**	**II-Objectives of Response to DMTs**
In all patients with SMA, the following clinical and contextual determinants should be considered when establishing therapeutic response objectives to DMTs:	In all patients with SMA, evaluation of response to DMTs should consider the following clinical and patient-centered objectives
(a) Baseline functional status, including the ability to achieve sitting and ambulation.	**(a) Respiratory, swallowing, and bulbar function**.
(b) SMA type (types I, II, III, and IV).	Within each of these evaluations, improvement should be considered a treatment response parameter.
(c) Patient age at treatment initiation.	In selected patients, stability of each of these functions may be considered an appropriate response parameter depending on response determinants such as SMA type, age, disease duration, and baseline functional level.
(d) Disease duration.	**(b) Quality of life**.
(e) Presence of scoliosis and, when applicable, performance of corrective surgery.	Within quality-of-life evaluation, improvement should be considered a treatment response parameter.
(f) Access to treatment.	**(c) Number of disease-related hospital admissions per year**.
	Within evaluation of hospital admissions per year, reduction should be considered a treatment response parameter.
	In selected patients, stability in the number of hospital admissions per year may be considered an appropriate response parameter depending on response determinants.
**III. Tools for Evaluation of Response to DMTs**	
In patients with SMA, validated tools selected according to age, functional level, and clinical domain should be used for response evaluation to DMTs:	
**Motor function**	
(a) CHOP-INTEND scale for evaluation of motor function in patients ≤24 months.	
(b) HFMSE and RULM scales for evaluation of motor function in patients older than 24 months who are non-ambulatory.	
(c) HFMSE scale and the 6-minute walk test (6MWT) for evaluation of motor function in patients older than 24 months who are ambulatory.	
(d) EK2 scale for evaluation of motor function in patients older than 4 years who are non-ambulatory.	
**Quality of life**	
(a) PedsQL neuromuscular module, caregiver-reported version, for evaluation of quality of life in patients between 2 and 18 years of age.	
(b) PedsQL neuromuscular module, patient-reported version, for evaluation of quality of life in patients between 5 and 18 years of age.	
**Respiratory function**	
(a) Spirometry with assessment of forced vital capacity (FVC), forced expiratory volume in the first second (FEV1), and FEV1/FVC ratio in patients aged 5 years and older.	
(b) Oximetry for evaluation of respiratory function in patients younger than 5 years.	
(c) Transcutaneous capnography for evaluation of respiratory function in patients younger than 5 years.	
**Bulbar function**	
(a) Videofluoroscopic swallow study for evaluation of bulbar function in patients with SMA.	
(b) Oral and Swallowing Abilities Tool (OrSAT) for evaluation of bulbar function in patients younger than 2 years of age.	
(c) Structured bulbar evaluation tools in patients older than 2 years, such as the SAFE (Swallowing Ability and Function Evaluation) scale, although validated Spanish-language versions remain limited.	
(d) EK2 scale (bulbar items) for evaluation of bulbar function in patients older than 2 years.	
**IV. Frequency of Evaluation of Response to DMTs**	**V. Initiation and Discontinuation of DMTs**
Evaluation of response to DMTs and decisions regarding continuation or discontinuation should be based on achievement of defined therapeutic goals, considering the following intervals:	**Initiation of DMTs is recommended in the presence of symptoms and objectively measurable functional impairment through motor and/or functional scales**.
(a) Every 12 to 18 months in patients who initiate treatment early.	(a) In adult patients with SMA diagnosis, DMT initiation is recommended as early as possible after a confirmed diagnosis of SMA, including in presymptomatic patients identified through newborn screening. In symptomatic patients, clinical symptoms and objectively measurable functional impairment should be documented as part of the baseline assessment.
(b) Every 18 to 24 months in patients who initiate treatment later.	(b) In indicated cases, DMTs should be initiated as rapidly as possible.
(c) Evaluation frequency should be individualized according to patient characteristics, particularly functional status, age, and disease duration.	**Discontinuation of DMTs should be considered on an individualized basis within a clinical and ethical framework in the following situations:**
	(a) In pediatric patients, when the treating team and ethics committee determine lack of clinically meaningful benefit.
	(b) In pediatric patients, in the presence of severe medication-related adverse events.
	(c) In pediatric patients with advanced-stage disease when minimal or absent expectations of meaningful change with treatment exist.
	(d) In adult patients, when the treating team determines lack of clinically meaningful benefit.
	(e) In adult patients, in the presence of severe medication-related adverse events.
	(f) In adult patients with advanced-stage disease (permanent ventilatory dependence, absence of voluntary movement).

It is recognized that therapeutic response is conditioned by the patient’s baseline status, including clinical phenotype, age at symptom onset, time elapsed until treatment initiation, and functional level at treatment onset, particularly regarding motor milestone acquisition such as sitting or ambulation. Contextual and orthopedic variables, including the presence of scoliosis, performance of corrective surgeries, and timely access to treatment, also influence outcomes. These factors determine the potential for improvement or stabilization and justify avoiding non-contextualized uniform criteria [8,22,41,42].

The panel emphasizes that motor evaluation remains central but must be interpreted according to natural history; in certain patients, sustained functional stability constitutes a clinically meaningful outcome. Additionally, systematic integration of non-motor domains (respiratory, bulbar/nutritional), quality of life, and reduction or stability of disease-related hospitalizations is recommended, given their impact on morbidity, mortality, and activities of daily living [22,42].

To objectively assess response, the use of validated tools selected according to age, functional level, and clinical domain is recommended, including CHOP-INTEND in children younger than 24 months, HFMSE and RULM in non-ambulatory patients, and HFMSE together with the 6MWT in ambulatory patients, as well as quality-of-life questionnaires such as the PedsQL neuromuscular module. In adults, the utility and limitations of motor and functional scales in longitudinal follow-up are emphasized [22,37,38,43].

Quality of life should be assessed across all age groups and functional levels using validated tools adapted to patient age and profile [44].

Regarding evaluation frequency, the panel proposes that decisions on treatment continuation or discontinuation should be based on achievement of therapeutic goals, with suggested intervals of 12–18 months for early treatment initiation and 18–24 months for later treatment initiation, individualized according to age, functional status, and disease duration.

Regarding initiation of DMTs, treatment is recommended upon the presence of symptoms and objectively measurable functional impairment through motor and/or functional scales, including adult patients when documented clinical involvement exists [8,9,10,11,12,13,14,15,16,17,18,19].

In settings where newborn screening is unavailable, treatment initiation should be considered from the appearance of first symptoms, given that indication and diagnosis remain fundamentally clinical, while scales serve as useful tools for longitudinal monitoring [45,46].

The panel recommends initiating DMTs as rapidly as possible, considering the impact of disease duration on response potential [8,9,10,11,12,13,14,15,16,17,18,19,27].

Finally, discontinuation of DMTs should be considered on an individualized basis within a clinical and ethical framework, particularly in cases of lack of clinically meaningful benefit, occurrence of severe drug-related adverse events, or advanced disease with minimal expectations for meaningful change (for example, permanent ventilatory dependence and absence of voluntary movement), in both pediatric and adult populations [27,28,47] (Table 4).

## 5. Discussion 

This Latin American consensus synthesizes recommendations for the diagnosis, multidisciplinary management, and evaluation of response to DMTs in SMA, integrating available evidence with regional experience and contextual information regarding real-world access across LATAM (Table 2, Tables 3.1,3.2,3.3,3.4 and 4, and Fig. 3). The paradigm shift generated by DMTs has substantially transformed the natural history of SMA, giving rise to new clinical trajectories and the emergence of previously undescribed phenotypes, redefining the disease as a chronic condition requiring longitudinal follow-up and individualized therapeutic goals, particularly when treatment is initiated early [8,9,10,11,12,13,14,15,16,17,18,19].

In patients treated early, the acquisition of previously unimaginable motor milestones has been observed, creating new challenges for healthcare systems, specialized medical teams, as well as for patients and their families, thus requiring dynamic adaptation of care models and multidisciplinary management strategies to these evolving clinical profiles [8,12,13,14,48]. More recently, new therapeutic strategies and dosing regimens under evaluation or approval in certain regulatory settings have been reported, reflecting the continuous evolution of SMA treatment [49,50,51,52].

In this context, longitudinal follow-up of individuals with SMA should not only focus on prevention and management of disease-associated complications, but also requires the systematic incorporation of standardized tools that allow objective, reproducible, and comparable evaluation of therapeutic response [1,22,27,47]. Within this evolving scenario, maintaining alignment with international standards of care while adapting implementation to the resource and access asymmetries characteristic of the region is critical [1,22,27,28,47].

A relevant finding of the regional survey was the marked heterogeneity in key components of the care process (Table 1). Although most respondents reported MLPA availability, only 35% indicated that this test was included within the public healthcare system, while the remainder depended on private funding or industry support. Similarly, access to at least one disease-modifying therapy was reported; however, important gaps persist in genetic confirmation timelines, absence of newborn screening in most countries, frequent delays between diagnosis and treatment initiation, as well as incomplete access to multidisciplinary units and home ventilation programs. These findings reinforce that DMT effectiveness depends in practice on a system that enables timely diagnosis, early referral, and continuous multidisciplinary support [22,42,45,46,53].

Consistent with the literature, this consensus emphasizes that response to DMTs should not be based exclusively on motor milestone gains, but rather on a domain-based, patient-centered evaluation that considers improvement or stability according to baseline determinants such as SMA type, age at onset, functional status, and disease duration [1,22,27]. This approach is particularly relevant in patients with greater motor impairment, in whom clinical stabilization may represent a clinically meaningful benefit compared with progressive natural history, and aligns with evidence from pivotal trials demonstrating variable response magnitudes according to treatment timing and clinical stage [8,10,11,12,13,48].

Stratification by functional level (non-sitters, sitters/non-ambulatory, and ambulatory patients) provides a practical structure for operationalizing realistic and comparable therapeutic goals. In non-sitters, integrating respiratory and bulbar domains into response definitions is essential given the impact of respiratory and swallowing compromise on morbidity, mortality, hospitalizations, and quality of life [1,22,27]. In sitters, preservation of previously attained abilities, particularly upper limb function, along with systematic monitoring of respiratory, bulbar/nutritional, and quality-of-life domains are critical components of therapeutic benefit beyond acquisition of new motor milestones [1,22,27]. In ambulatory patients, slowing of deterioration, assessment of endurance and performance, and identification of falls and fatigue as indicators of functional progression allow more sensitive adjustment of rehabilitation and follow-up, particularly during adolescence and early adulthood [1,22,27].

The recommendation to use validated tools according to age, domain, and functional status aims to reduce clinical variability and facilitate reproducible decision-making. Inclusion of CHOP-INTEND in children younger than 24 months, HFMSE/RULM in non-ambulatory patients, and 6MWT plus HFMSE in ambulatory patients is consistent with their widespread use in clinical research and practice, and supports detection of subtle but clinically relevant changes when the principal objective is stabilization or slowing of progression [37,38,43,47,54,55]. Likewise, emphasis on instruments measuring bulbar function and objective respiratory metrics contributes to a more comprehensive therapeutic impact assessment [22,47].

Regarding newborn screening, its implementation emerges as a regional public health priority. Pilot experiences and management algorithms for infants identified through newborn screening support both its feasibility and its potential to maximize therapeutic benefit by enabling presymptomatic or very early treatment, which may ultimately determine whether a patient achieves ambulation [45,46].

In parallel, this consensus proposes a pragmatic framework for decision-making regarding continuation or discontinuation of DMTs, incorporating clinical and ethical criteria (clinically meaningful benefit, severe adverse events, advanced disease), which is particularly valuable in settings with eligibility committees and access restrictions [1,22,26,53,56].

This work has limitations. The survey reflects professional perceptions and may be subject to selection bias, overrepresentation of urban settings, and variability in understanding of local administrative pathways. Additionally, although the consensus methodology was structured, it remains dependent on panel composition and available evidence; in areas with limited data, some recommendations represent the best possible synthesis between evidence and regional applicability. Additionally, the survey did not systematically capture country-level distinctions between regulatory approval, public or private reimbursement, effective patient access, and specific eligibility restrictions for individual DMTs, limiting more granular comparisons of treatment access across countries. Finally, access to DMTs may differ between pediatric and adult patients across Latin American healthcare systems, and the inclusion of respondents caring exclusively for adults may have contributed to heterogeneity in reported treatment access [25,42,46,53].

Furthermore, although psychological support for patients and families, as well as genetic counseling, are fundamental components of comprehensive SMA care, these aspects were not specifically addressed in this consensus because they are not exclusive to SMA and should instead be structurally integrated into multidisciplinary care programs for neuromuscular diseases more broadly.

Additionally, the absence of adequately structured referral centers in several regional healthcare systems, together with organizational and resource heterogeneity among countries, represents an important limitation that may influence interpretation and generalizability of findings. Nevertheless, the strength of this document lies in its practical orientation, alignment with international standards, and explicit integration of implementation barriers within LATAM.

Finally, the results identify priority areas for future research and implementation: expansion of newborn screening, reduction of diagnostic and therapeutic delays, strengthening of multidisciplinary networks, standardization of domain-based metrics, and generation of prospective regional data (registries) to evaluate effectiveness, safety, indirect costs, and patient-centered outcomes in real-world care settings [26,46,47,53].

In conclusion, this Latin American consensus proposes practical recommendations for diagnosis, multidisciplinary management, and evaluation of response to DMTs in SMA, adapted to the LATAM context and aligned with international evidence and standards of care. The recommendations emphasize longitudinal domain-based evaluation (motor, respiratory, bulbar/nutritional, quality of life, and hospitalizations) and stratification by functional level to define realistic therapeutic goals, including clinical stability as a relevant outcome in selected subgroups. The regional survey demonstrates persistent gaps in newborn screening, access timelines, and homogeneous availability of multidisciplinary resources, underscoring the need for regional strategies to improve timely diagnosis, early DMT initiation, and standardized follow-up.

Collectively, this first Latin American consensus constitutes an initial milestone and a starting point for the development of a collaborative SMA agenda in the region. This process has integrated the perspectives and expertise of specialists from 15 countries, consensually identifying priority gaps and opportunities for improvement across the continuum of care, from early diagnosis to longitudinal treatment response monitoring. This effort establishes the foundation for consolidating a regional working group aimed at advancing multidisciplinary care standardization, harmonized metric implementation, and generation of region-specific evidence to support both clinical and healthcare policy decision-making in LATAM.
