# Supplementary material for: Consensus for the Diagnosis and Treatment of Patients With Spinal Muscular Atrophy (SMA) in Latin America
Source: Rev Neurol. 2026 Sep 23;81(9):53782. doi: 10.31083/RN53782 (PMC13653183; doi:10.31083/RN53782)
Supplement: Supplementary file 1 [file 1576-6578-81-9-53782-s1.zip › en-Supplementary Material 1.docx]

**SMA Availability and Access Questionnaire**

**Q1 In which country do you practice medicine?**

Free-text response

**Q2 In which city is the medical site where you spend most of your time seeing patients located?**

Free-text response

**Q3 Which of the following best describes the location where you practice medicine?**

• Rural (<50,000 inhabitants)

• Small city (50,000–100,000 inhabitants)

• Large city (100,000–1 million inhabitants)

• Urban area (>1 million inhabitants)

**Q4 Which age group do you primarily treat?**

• Children (0–17 years of age)

• Adults (18+ years of age)

• Both children and adults

**Section 2: Diagnostic Testing / The following questions focus on your experience diagnosing SMA (spinal muscular atrophy) and the availability of diagnostic testing for SMA in your medical practice.**

**Q5 Do you have access to MLPA (Multiplex Ligation-dependent Probe Amplification) for the diagnosis of SMA?**

- Yes
- No
- I don't know

**Q6 If you have access to MLPA for diagnosis, approximately how long does it take to receive the test result?**

Two weeks.

Four weeks.

Four to eight weeks.

More than eight weeks.

I don't know.

**Q7 To perform MLPA, which of the following options do you use? (You may select more than one option.)**

- The test is available within your country's public healthcare system.
- You obtain the test through private healthcare.
- You request support from the pharmaceutical industry to cover the cost of the test.

**Q8 Is newborn screening for SMA available in your country?**

As part of the national newborn screening program.

As a pilot study or as a proposed program planned for future implementation.

SMA newborn screening is not currently planned.

**Section 3: Treatment / The following questions focus on your experience treating patients with SMA, treatment units, and the availability of pharmacological treatments for disease management.**

**Q9 Are there multidisciplinary units in your country with experience in the care of patients with SMA?**

- Yes
- No
- I don't know

**Q10 Approximately what proportion of patients with SMA in your country have access to multidisciplinary disease management (respiratory care, rehabilitation, swallowing care)?**

100% of patients

80% of patients

50% of patients

Less than 20% of patients

**Q11 In your country, do patients with SMA have access to a home invasive and non-invasive mechanical ventilation program?**

- Yes.
- No
- I don't know

**Q12 Do you have access to disease-modifying treatments for spinal muscular atrophy?**

- Yes
- No
- I don't know

**Q13 Which of the approved therapies for SMA are currently available to you? (You may select more than one option.)**

Onasemnogene

Risdiplam

Nusinersen

I do not have access to therapies for SMA

**Q14 Do you use other medications for the management of patients with SMA?**

Yes

No

If your answer is Yes, please describe (free text).

**Q15 Access to these therapies is:**

Private.

Public.

Public and private.

I don't know.

**Q16 Approximately how long does it take for a patient newly diagnosed with SMA in your country to receive disease-modifying treatment?**

Less than two weeks.

Two to four weeks.

One to six months.

More than 6 months.

I don't know.

Other

Please describe other.

**Q17 Do you have a health committee that decides whether your patient is eligible to receive treatment?**

- Yes
- No
- I don't know
