# Supplementary material for: Consensus for the Diagnosis and Treatment of Patients With Spinal Muscular Atrophy (SMA) in Latin America
Source: Rev Neurol. 2026 Sep 23;81(9):53782. doi: 10.31083/RN53782 (PMC13653183; doi:10.31083/RN53782)
Supplement: Supplementary file 1 [file 1576-6578-81-9-53782-s1.zip › es-Supplementary Material 1.docx]

**Cuestionario disponibilidad y acceso AME**

**Q1 ¿En qué país practica medicina?**

Formato de respuesta Libre

**Q2 ¿En qué ciudad se encuentra el sitio médico donde Ud. pasa la mayoría del tiempo atendiendo a los pacientes?**

Formato de respuesta Libre

**Q3 ¿Cuál de las siguientes respuestas mejor describe el sitio dónde Ud. practica medicina?**

• Rural (< 50,000 habitantes)]

• Ciudad pequeña (50,000 – 100,000 habitantes)

• Ciudad grande (100,000 – 1 millón de habitantes)

• Área urbana (> 1 millón de habitantes)

**Q4 ¿qué grupo de edad trata Ud. principalmente?**

• Niños (edad 0-17 años)]

• Adultos (edad 18+ años)

• Tanto niños como adultos

**Sección 2: Estudios de Diagnóstico / Las siguientes preguntas se enfocarán en su experiencia diagnosticando AME (atrofia muscular espinal) y la disponibilidad de estudios de diagnóstico de AME en su práctica médica**

**Q5 ¿Cuenta con la técnica MLPA (Multiplex Ligation-dependent Probe Amplification) para diagnóstico de AME?**

- Sí
- No
- No sé

**Q6 En caso de contar con la técnica de MLPA para el diagnóstico ¿En cuánto tiempo tiene el resultado del test aproximadamente?**

Dos semanas.

Cuatro semanas.

Cuatro a ocho semanas.

Más de ocho semanas

No lo sabe

**Q7 Para realizar la técnica MLPA usted (puede elegir más de una opción)**

- Cuenta con el estudio dentro del sistema público de salud de su país.
- Lo envía de manera privada
- Se lo solicita apoyo a la industria farmacéutica para costear el estudio

**Q8 En su país existe Cribado de pacientes con AME?**

Dentro del programa nacional de cribado.

Como estudio piloto o como propuesta que se iniciará a futuro.

No esta contemplado por el momento tener cribado de AME

**Sección 3. Tratamiento /Las siguientes preguntas se enfocarán en su experiencia tratando a pacientes con AME, unidades de tratamientos y la disponibilidad de los tratamientos farmacológicos para el manejo de la enfermedad.**

**Q9 ¿Existe en su país unidades multidisciplinarias con experiencia en la atención de pacientes con AME?**

- Sí
- No
- No lo sé

**Q10 ¿Qué proporción de los pacientes con AME en su país cuentan con acceso a manejo multidisciplinario la enfermedad aproximadamente (respiratorio, rehabilitación, deglución)?**

100% de pacientes

80% de pacientes

50% de pacientes

Menos de 20% de pacientes

**Q11 ¿En su país, cuentan con un programa de ventilación mecánica invasiva y no invasiva domiciliaria los pacientes con AME?**

- Sí.
- No
- No lo sé

**Q12 ¿Cuenta con acceso a tratamientos modificadores de la enfermedad para atrofia muscular espinal?**

- Sí
- No
- No lo sé

**Q13 ¿Con cuáles de las terapias aprobadas para AME dispone actualmente? (puede elegir más de una opción)**

Onasemnogene

Risdiplam

Nusinersen

No cuento con terapias para AME

**Q14 Utiliza otros fármacos para el manejo de pacientes con AME?**

Si

No

En caso de que su respuesta sea Si, describa si (texto libre)

**Q15 El acceso de estas terapias es de forma:**

Privada.

Pública.

Pública y privada.

No lo sé

**Q16 ¿Cuánto tiempo toma aproximadamente para que un paciente con recién diagnóstico de AME en su país reciba tratamiento modificado de la enfermedad?**

Menos de dos semanas.

Dos a cuatro semanas.

Dos a seis meses.

Más de 6 meses.

No lo sé

Otro

Describa otro

**Q17 ¿Cuenta con un comité de salud que decide si su paciente es candidato para recibir tratamiento?**

- Sí
- No
- No lo sé
