# Supplementary material for: Consensus for the Diagnosis and Treatment of Patients With Spinal Muscular Atrophy (SMA) in Latin America
Source: Rev Neurol. 2026 Sep 23;81(9):53782. doi: 10.31083/RN53782 (PMC13653183; doi:10.31083/RN53782)
Supplement: Translation

# Translation: Consenso para el Diagnóstico y Tratamiento para Pacientes con Atrofia Muscular Espinal (AME) en América Latina

**Published:** 2026-09-23

**Authors:** Andrés E. Nascimento Osorio, Graciela Barros Acevedo, María de los Ángeles Beytía, Edna J. Bobadilla Quesada, Sandra M. Castellar-Leones, Claudia Castiglioni, Marco J. Casartelli Galeano, Guilca Contreras Caicedo, María Fernanda Cordero Molina, Rosa E. Escobar Cedillo, Francisco Espinel, Nicholas Earle, Carlos A. Franco Toñanez, Henry H. Galvez Quiñonez, Juliana Gurgel-Giannetti, Alfonso P. Gutiérrez Mata, María C. Hervias Ruz, Javier E. Linzoain, Mariela A. Lucero, Conrado Medici, Sandra C. Mesa Restrepo, María E. Meza Cano, Soledad Monges, Leticia M. Munive Baez, Lourdes I. Núñez Antelo, Rogelio R. Odales Ibarra, Verónica D. Artiga-Rodríguez, Eva S. Pérez Almengor, Peggy C. Martínez Esteban, Diana Ramírez-Montaño, Rodrigo Moreno-Salgado, Matilde Ruiz García, Verónica Sáez Galaz, Edwin S. Vargas Cañas, Jorge A. Bevilacqua, Edmar Zanoteli, Carol J. Zuniga García, María R. Gueçaimburu, Mario Gutiérrez-Saenz, Jesús A. Armijo Gómez

**Affiliations:** ^60^Unidad de Neuromuscular, Hospital Sant Joan de Déu; Group of Applied Research in Neuromuscular Diseases, Institut de Recerca Sant Joan de Déu, 08950 Barcelona, España; ^61^Center for the Biomedical Research on Rare Diseases (CIBERER), ISCIII, 08036 Barcelona, España; ^62^Centro Hospitalario Pereira Rossell, Instituto de Neurología, 11600 Montevideo, Uruguay; ^63^Teletón Uruguay, 11600 Montevideo, Uruguay; ^64^Sección de Neurología Pediátrica, División de Pediatría, Escuela de Medicina, Pontificia Universidad Católica de Chile, 8331150 Santiago, Chile; ^65^Unidad de Neurología Pediátrica, Servicio de Pediatría, Hospital Dr. Sótero del Río, 8150215 Santiago, Chile; ^66^Departamento de Pediatría, Fundación Hospital de la Misericordia (HOMI), 111411 Bogotá, Colombia; ^67^Hospital Universitario San Ignacio, 110231 Bogotá, Colombia; ^68^Departamento de Medicina Física y Rehabilitación, Facultad de Medicina, Universidad Nacional de Colombia, 111321 Bogotá, Colombia; ^69^Hospital Universitario Nacional de Colombia, 111321 Bogotá, Colombia; ^70^Departamento de Pediatría, Clínica MEDS, 7510155 Santiago, Chile; ^71^Escuela de Medicina, Universidad Finis Terrae, 7501015 Santiago, Chile; ^72^Hospital General Pediátrico Niños de Acosta Ñu, Ministerio de Salud Pública y Bienestar Social, 2160 San Lorenzo, Paraguay; ^73^Servicio de Neuropediatría, Centro Médico Docente La Trinidad, 1080 Caracas, Venezuela; ^74^Neurología Infantil, Clínica Monte Sinaí, 010107 Cuenca, Ecuador; ^75^Clínica Latinoamericana, 010107 Cuenca, Ecuador; ^76^Instituto Nacional de Rehabilitación “Luis Guillermo Ibarra Ibarra” (INR-LGII), 14389 Ciudad de México, México; ^77^Unidad de Neurología Pediátrica, Servicio de Pediatría, Hospital Metropolitano, 170135 Quito, Ecuador; ^78^Departamento de Neurología y Neurocirugía, Clínica Dávila, Santiago, Chile, and Corporación de Investigación de Neurología de Santiago (CINSAN), 8431657 Santiago, Chile; ^79^Departamento de Neuropediatría, Hospital General Pediátrico Niños de Acosta Ñu, 2160 San Lorenzo, Paraguay; ^80^Centro GALO, 01015 Ciudad de Guatemala, Guatemala; ^81^Hospital Regional de Occidente HRO, 09001 Ciudad de Guatemala, Guatemala; ^82^Department of Pediatrics, Faculdade de Medicina da Universidade Federal de Minas Gerais (UFMG), 30130-100 Belo Horizonte, Brazil; ^83^Hospital Nacional de Niños de Costa Rica “Dr. Carlos Sáenz Herrera”, 10101 San José, Costa Rica; ^84^Neurología Infantil, Clínica MEDS, 7510155 Santiago, Chile; ^85^Escuela de Kinesiología, Facultad de Medicina, Universidad de los Andes, 7620001 Santiago, Chile; ^86^Servicio de Neurología Pediátrica, Clínica Universitaria Reina Fabiola, X5000 Córdoba, Argentina; ^87^Hospital Infantil Municipal de la Ciudad de Córdoba, X5000 Córdoba, Argentina; ^88^Servicio de Neurología Infantil, Hospital de Niños Sor María Ludovica, 1900 La Plata, Buenos Aires, Argentina; ^89^Hospital Pablo Tobón Uribe, 050031 Medellín, Colombia; ^90^Hospital CHRISTUS MUGUERZA Alta Especialidad, 64060 Monterrey, México; ^91^Instituto Tecnológico de Monterrey, 64849 Monterrey, México; ^92^Servicio de Neurología, Hospital de Pediatría S.A.M.I.C. “Prof. Dr. Juan P. Garrahan”, 1245 Buenos Aires, Argentina; ^93^Servicio de Neurología, Hospital Médica Sur, 14050 Ciudad de México, México; ^94^Instituto Nacional de Pediatría, 04530 Ciudad de México, México; ^95^VITAE Medicina y Diagnóstico, 10260 Santa Cruz de la Sierra, Bolivia; ^96^Caja Petrolera de Salud, 10260 Santa Cruz de la Sierra, Bolivia; ^97^Hospital Pediátrico Baca Ortiz, 170136 Quito, Ecuador; ^98^Universidad de las Américas (UDLA), 170125 Quito, Ecuador; ^99^Servicio de Neuropediatría, Hospital Nacional de Niños Benjamín Bloom, 1101 San Salvador, El Salvador; ^100^Clínica Neuroplastickids, 1101 San Salvador, El Salvador; ^101^Hospital del Niño, Dr. José Renán Esquivel, 0816-02189 Ciudad de Panamá, Panamá; ^102^Instituto Nacional de Salud del Niño San Borja, 15036 Lima, Perú; ^103^Unidad de Medicina Genómica y Genética, Clínica Imbanaco Grupo Quirón Salud, 760042 Cali, Colombia; ^104^Departamento de Morfología, Facultad de Salud, Universidad del Valle; Hospital Universitario del Valle Evaristo García ESE, 760032 Cali, Colombia; ^105^Fundación Padrino Todos Santos A.C., 23300 Todos Santos, Baja California Sur, México; ^106^Servicio de Neuropsiquiatría Infantil, Hospital Clínico San Borja Arriarán, 8320000 Santiago, Chile; ^107^Clínica Universidad de los Andes, 7620001 Santiago, Chile; ^108^Clínica de Enfermedades Neuromusculares, Instituto Nacional de Neurología y Neurocirugía “Manuel Velasco Suárez”, 14269 Ciudad de México, México; ^109^Centro de Neurociencias, Hospital Ángeles del Pedregal, 01900 Ciudad de México, México; ^110^Departamento de Neurología y Neurocirugía, Hospital Clínico Universidad de Chile & Departamento de Anatomía y Medicina Legal, Facultad de Medicina, Universidad de Chile, 8380453 Santiago, Chile; ^111^Department of Neurology, Faculdade de Medicina, Universidade de São Paulo (FMUSP), 01246-903 São Paulo, Brazil; ^112^Hospital Mario Catarino Rivas, Servicio de Neurología Pediátrica, 21104 San Pedro Sula, Cortés, Honduras; ^113^Hospital del Valle, 21102 San Pedro Sula, Cortés, Honduras; ^114^Equipo de Enfermedades Raras, CRENADECER, BPS, 11200 Montevideo, Uruguay; ^115^Neurología, Hospital Clínica Bíblica, 10101 San José de Costa Rica, Costa Rica; ^116^Caja Costarricense del Seguro Social, 10104 San José de Costa Rica, Costa Rica; ^117^Hospital General con Especialidades Juan María Salvatierra, 23000 La Paz, México; ^118^Neurología Pediátrica & Patología Neuromuscular, 23000 La Paz, México

**Keywords:** atrofia muscular espinal, atrofias musculares espinales infantiles, gen de supervivencia de la neurona motora 1 (*SMN1*), gen de supervivencia de la neurona motora 2 (*SMN2*), nusinersen, risdiplam, terapia génica, pesquisa neonatal, América Latina, consenso

## Abstract

**Antecedentes:**

La atrofia muscular espinal ligada al cromosoma 5q (AME 5q) es una enfermedad neuromuscular genética autosómica recesiva causada por mutaciones en el gen *SMN1*, que generan deficiencia de la proteína de supervivencia de la neurona motora (SMN) y un espectro clínico variable influido por el número de copias de *SMN2*. La introducción de terapias modificadoras de la enfermedad (TME) ha transformado su evolución, requiriendo diagnóstico oportuno, abordaje multidisciplinario y evaluación longitudinal de la respuesta. En América Latina (LATAM), persisten brechas relevantes y heterogeneidad en el acceso a diagnóstico genético, pesquisa neonatal, TME y seguimiento especializado. Desarrollar un consenso latinoamericano con recomendaciones para el diagnóstico, tratamiento integral (respiratorio, deglución/nutrición, rehabilitación y TME) y evaluación de la respuesta a TME en pacientes con AME, incorporando información contextual sobre acceso en la región.

**Métodos:**

Se conformó un panel regional de expertos que trabajó entre 2025 y 2026 mediante un proceso de consenso formal con rondas sucesivas de cuestionarios y discusión estructurada. Se definió acuerdo con ≥70% de concordancia. Adicionalmente, se realizó una encuesta regional para evaluar el acceso a recursos diagnósticos y terapéuticos.

**Resultados:**

Participaron 40 profesionales de 15 países. Mientras que el 90% de los encuestados informó tener acceso a pruebas genéticas (MLPA), solo el 35% indicó que estas estaban disponibles dentro del sistema público de salud; también se señalaron el acceso privado y el apoyo de la industria farmacéutica como vías para realizar las pruebas. Ningún país participante informó la implementación a nivel nacional del cribado neonatal para AME; el 22,5% de los encuestados informó la existencia de programas piloto o propuestas de implementación futura. El acceso a al menos una terapia modificadora de la enfermedad (TME) fue informado por el 92,5% de los encuestados; sin embargo, los retrasos entre el diagnóstico y el inicio del tratamiento fueron frecuentes y continúan siendo un desafío importante en la región. Se consensuaron recomendaciones diagnósticas, manejo multidisciplinario estratificado por nivel funcional y criterios de evaluación de respuesta basados en dominios motores y no motores con herramientas validadas y seguimiento individualizado.

**Conclusiones:**

Este consenso proporciona un marco práctico y adaptable para optimizar la atención de la AME en LATAM, promoviendo decisiones clínicas longitudinales y centradas en el paciente.

## 1. Introducción

La atrofia muscular espinal (AME) es un grupo heterogéneo de enfermedades neuromusculares hereditarias, de baja prevalencia, caracterizadas por la degeneración progresiva de las motoneuronas del asta anterior de la médula espinal y de los núcleos motores bulbares del troncoencéfalo, lo que conduce a debilidad y atrofia muscular de distribución variable, con frecuente compromiso respiratorio y bulbar, y elevada mortalidad en las formas más graves y de inicio temprano [1,2].

Desde el punto de vista genético, la AME se clasifica en dos grandes categorías: la AME ligada al cromosoma 5q (AME 5q), que representa aproximadamente el 90–95% de los casos y presenta una incidencia estimada de 1 por cada 5000–10.000 nacidos vivos [3,4].

La AME 5q está causada, en la mayoría de los casos, por deleciones homocigotas del exón 7 del gen *SMN1* y, en menor proporción, por variantes puntuales, lo que condiciona una reducción crítica de la proteína de supervivencia de la neurona motora (SMN) [5,6]. El gen parálogo *SMN2* actúa como el principal modificador del fenotipo, ya que genera cantidades limitadas de proteína funcional; así, un mayor número de copias se asocia con presentaciones clínicas más leves, configurando un espectro continuo que abarca desde formas de inicio prenatal hasta manifestaciones en la edad adulta [3,6,7]. En el presente consenso latinoamericano, nos centraremos específicamente en la AME 5q, abordando de manera integral los aspectos diagnósticos, terapéuticos y de manejo multidisciplinario en el contexto actual.

Durante la última década, el abordaje terapéutico de la AME ha experimentado un cambio sustancial con la introducción de terapias modificadoras de la enfermedad dirigidas a restaurar la producción de proteína SMN. Ensayos clínicos pivotales han demostrado que tratamientos como nusinersen, risdiplam y la terapia génica con onasemnogene abeparvovec mejoran la supervivencia, la función motora y los hitos del desarrollo, especialmente cuando se administran en las fases presintomáticas de la enfermedad [8,9,10,11,12,13,14,15,16,17,18,19]. Estos avances han transformado la AME de una enfermedad rápidamente progresiva y frecuentemente letal en una patología crónica que requiere seguimiento a largo plazo [1,8,20].

No obstante, la atención de las personas con AME no se limita únicamente a terapias modificadoras de la enfermedad (TME) sino que requiere un abordaje integral [1,21,22]. En este sentido, las guías internacionales de manejo destacan la necesidad de un abordaje integral y multidisciplinario que incluya el cuidado respiratorio, la evaluación y manejo de la deglución y la nutrición, la rehabilitación motora, el seguimiento ortopédico y la monitorización sistemática de la función motora y respiratoria [1,21,22]. La integración de estas intervenciones son fundamentales para optimizar los resultados clínicos, prevenir complicaciones y mejorar la calidad de vida de los pacientes [21,22,23] (Fig. 1).

**Fig. 1.** **Componentes del abordaje integral y multidisciplinario en pacientes con atrofia muscular espinal**. Ref: Esquema conceptual del manejo integral de la atrofia muscular espinal, que incluye diagnóstico temprano, terapia modificadora de la enfermedad (TME) y cuidado multidisciplinario continuo (respiratorio, nutricional, deglución, rehabilitación, seguimiento ortopédico y monitorización funcional), necesarios para optimizar los resultados clínicos y la calidad de vida. AME, atrofia muscular espinal.

En América Latina (LATAM), la implementación de los estándares internacionales de cuidado para la AME enfrenta desafíos significativos, derivados de la heterogeneidad de los sistemas de salud, las diferencias regulatorias entre países y la disponibilidad variable de diagnóstico genético confirmatorio. A ello se suman barreras geográficas y de acceso a centros de referencia y programas de tamizaje neonatal, así como inequidades en el acceso a TME y a equipos multidisciplinarios con experiencia y recursos para el manejo integral de estos pacientes [24,25]. Estas disparidades condicionan retrasos diagnósticos, variabilidad en las decisiones terapéuticas y dificultades en el seguimiento longitudinal de los pacientes con AME en la región [24,26].

En consecuencia, resulta necesario desarrollar recomendaciones en la región que, manteniendo la alineación con la evidencia científica y los consensos internacionales principalmente en países desarrollados, contemplen las particularidades del contexto latinoamericano y sirvan como herramienta práctica para la toma de decisiones clínicas [1,27,28].

El presente consenso latinoamericano tiene como objetivo primario proveer recomendaciones para el diagnóstico, el tratamiento integral (incluyendo los aspectos respiratorios, deglutorios, de rehabilitación motora y TME), el monitoreo y la evaluación de la respuesta al tratamiento en pacientes con AME en LATAM, incorporando además información sobre el acceso a la atención y a las TME en la región como objetivo secundario.

## 2. Metodología

Un panel regional de expertos en enfermedades neuromusculares con experiencia en el diagnóstico, tratamiento y seguimiento longitudinal de pacientes con AME provenientes de distintos países de Latinoamérica se reunió durante 2025 y 2026 en diversas oportunidades, vía virtual, para llevar adelante el objetivo planteado a través del desarrollo de un consenso formal. Los expertos fueron identificados e invitados por los dos coordinadores del consenso (AENO y JAAG) sobre la base de su reconocida experiencia clínica en AME y su participación activa en el cuidado y seguimiento de pacientes con la enfermedad, procurando además una amplia representación geográfica de América Latina. El panel incluyó médicos de especialidades relevantes, incluyendo genética, todos activamente involucrados en la atención y manejo de pacientes con AME. El grupo de expertos estuvo coordinado por AENO y JAAG, quienes lideraron el proyecto desde su concepción hasta la instancia final. Para concretar el consenso se siguió la metodología de ronda de cuestionarios y discusión de acuerdos, desacuerdos de escenarios y situaciones clínicas [29]. Operativamente se realizó una reunión virtual para establecer los objetivos y alcances del consenso, posteriormente una primera, segunda y tercera ronda de cuestionarios vía virtual con los expertos y finalmente dos encuentros virtuales para la revisión y discusión de las recomendaciones generadas. Al momento de recibir el cuestionario, cada involucrado fue provisto de bibliografía complementaria para su revisión que le permitiese elaborar una respuesta más allá de su experiencia a cada afirmación propuesta [29]. A priori, se definió la presencia de acuerdo cuando ≥70% de los participantes respondían de forma homogénea; incertidumbre cuando entre el 40% y el 69% respondían de forma homogénea; y ausencia de acuerdo cuando menos del 40% respondían de forma homogénea. Las afirmaciones que no alcanzaron acuerdo durante la primera ronda fueron revisadas en cuanto a su claridad y aplicabilidad, reformuladas cuando fue necesario, complementadas con evidencia científica adicional y reevaluadas en las rondas posteriores. Las afirmaciones que permanecieron sin consenso luego de las rondas iterativas fueron discutidas por el panel completo; cuando finalmente no fue posible alcanzar consenso, no fueron presentadas como recomendaciones formales del consenso [29]. Las instancias de desarrollo de las afirmaciones se estratificaron en recomendaciones referentes a: (A) diagnóstico; (B) tratamiento (tratamiento respiratorio, deglución, rehabilitación, TME); (C) respuesta a TME en pacientes con AME. En este documento, el término TME incluye nusinersen, risdiplam y la terapia génica con onasemnogene abeparvovec. Los principales métodos estadísticos utilizados fueron medidas de tendencia central y dispersión: media, mediana, moda, máximo, mínimo y desvío estándar.

Una vez definidas las recomendaciones, se avanzó con la redacción del documento que fue posteriormente circulado entre los mismos para su acuerdo final y comunicación.

Secundariamente, con el objetivo de complementar las recomendaciones del consenso con información contextual sobre el acceso real al diagnóstico y tratamiento de la AME en LATAM, se desarrolló una encuesta específica dirigida a los participantes del consenso. La encuesta fue diseñada por el grupo coordinador y posteriormente validada por el grupo de trabajo, con el propósito de evaluar aspectos relacionados con la práctica clínica, la disponibilidad de recursos diagnósticos y terapéuticos, y las principales barreras de acceso en los diferentes contextos asistenciales. El cuestionario fue elaborado originalmente en idioma español y no se realizaron traducciones a otros idiomas. Previo a su implementación definitiva, el instrumento fue sometido a un proceso de validación piloto en dos instancias sucesivas. Cada prueba piloto incluyó la participación de cuatro médicos con experiencia en AME, quienes evaluaron la claridad de las preguntas, la pertinencia de los dominios explorados y la factibilidad de su aplicación [30,31]. Sobre la base de los comentarios recibidos, se realizaron ajustes en la redacción y organización de los ítems hasta obtener la versión final del cuestionario. La encuesta final estuvo estructurada para recopilar información en los siguientes dominios:

(a) características sociodemográficas y profesionales de los encuestados;

(b) disponibilidad de herramientas para el diagnóstico de la AME; y

(c) disponibilidad y condiciones de acceso a TME para la AME. Las respuestas de la encuesta fueron analizadas a nivel individual y no se agruparon para asignar clasificaciones oficiales por país; por lo tanto, las respuestas diferentes de participantes pertenecientes a un mismo país se mantuvieron tal como fueron reportadas. Las versiones finales de los cuestionarios utilizados se incluyen como **Material Suplementario 1**. Los resultados de la encuesta deben interpretarse como descriptivos y no necesariamente representativos de toda la región

## 3. Resultados

Durante el período septiembre-octubre de 2025, participaron 40 expertos provenientes de 15 países de Latinoamérica: Argentina, Bolivia, Brasil, Chile, Colombia, Costa Rica, Ecuador, El Salvador, Honduras, México, Panamá, Paraguay, Perú, Uruguay y Venezuela (Tabla 1, Fig. 2). La mayoría de los encuestados ejercía su práctica clínica en áreas urbanas de más de un millón de habitantes (85,0%), mientras que el 12,5% lo hacía en ciudades grandes (100.000–1 millón de habitantes) y el 2,5% en ciudades pequeñas. En cuanto a la población atendida, el 67,5% refirió tratar principalmente pacientes pediátricos, el 22,5% tanto niños como adultos y el 10,0% exclusivamente adultos (Tabla 1). Respecto a la disponibilidad de herramientas diagnósticas, el 90,0% de los encuestados informó contar con acceso a la técnica de MLPA para el diagnóstico de la AME. El tiempo estimado para obtener el resultado del estudio fue de ≤4 semanas en el 80,0% de los casos, mientras que el 15,0% reportó demoras de entre 4 y 8 semanas y el grupo restante demoras mayores a 8 semanas. En relación con la forma de acceso al estudio genético, el 35,0% indicó que se encuentra disponible dentro del sistema público de salud, el 35,0% lo envía de manera privada y el 30,0% solicita apoyo a la industria farmacéutica para su realización. En cuanto al cribado neonatal para AME, el 77,5% de los participantes reportó que no está contemplado actualmente en su país, mientras que el 22,5% refirió su existencia como estudio piloto o propuesta futura. En lo referente al tratamiento integral, el 77,5% de los encuestados informó la existencia de unidades multidisciplinarias con experiencia en la atención de pacientes con AME en su país. Sin embargo, solo el 12,5% estimó que el 100% de los pacientes accede a manejo multidisciplinario, mientras que el 45,0% refirió un acceso aproximado del 80%, el 20,0% del 50% y el 22,5% menos del 20% de los pacientes (Tabla 1, Fig. 3). La disponibilidad de programas de ventilación mecánica domiciliaria, invasiva o no invasiva, fue reportada por el 57,5% de los participantes. Respecto al acceso a TME, el 92,5% de los encuestados indicó disponer de al menos una terapia aprobada para AME. El 7,5% indicó no contar con terapias modificadoras disponibles. El acceso a estos tratamientos se describió mayoritariamente como público y privado en el 65,0%, exclusivamente público en el 27,5% y exclusivamente privado en el 5,0%. El tiempo estimado desde el diagnóstico hasta el inicio de la TME fue de ≤4 semanas en el 25,0% de los casos, mientras que el 42,5% reportó demoras de entre 1 y 6 meses, y el 20,0% demoras mayores a 6 meses. Finalmente, el 55,0% de los encuestados informó la existencia de un comité de salud encargado de decidir la elegibilidad de los pacientes para recibir tratamiento.

**Tabla 1.** **Características de los encuestados y acceso al diagnóstico y tratamiento de la AME**.

Dominio	Variable	n	%
Características de los encuestados	Países participantes	15	—
Características de los encuestados	Profesionales participantes	40	100
Características de los encuestados	Práctica en área urbana (>1 millón)	34	85,0
Características de los encuestados	Atención principalmente pediátrica	27	67,5
Diagnóstico	Acceso a MLPA	36	90,0
Diagnóstico	Resultado MLPA ≤4 semanas	32	80,0
Diagnóstico	Cribado neonatal no disponible	31	77,5
Tratamiento integral	Unidades multidisciplinarias disponibles	31	77,5
Tratamiento integral	Acceso ≥80% a manejo multidisciplinario	23	57,5
TME	Acceso a ≥1 TME	37	92,5
TME	Acceso público y privado	26	65,0
TME	Inicio de tratamiento 1–6 meses	17	42,5

TME, terapia modificadora de la enfermedad; MLPA, amplificación de sondas dependiente de ligación múltiple.

**Fig. 2.** **Distribución geográfica de los expertos participantes en el consenso**. Distribución geográfica de los expertos participantes en Latinoamérica. El mapa muestra los 15 países latinoamericanos representados en el proceso de consenso y el número (n) de expertos participantes por país. Un total de 40 expertos participaron en el consenso.

**Fig. 3.** **Panorama del acceso a la atención de la atrofia muscular espinal en América Latina: indicadores claves**. Ref: Mapa conceptual de la heterogeneidad en el acceso al diagnóstico y tratamiento de la atrofia muscular espinal (AME) en América Latina. Representación basada en resultados agregados de encuesta regional (n = 40 profesionales, 15 países). Colores tipo semáforo: verde = alta disponibilidad; amarillo = disponibilidad intermedia; rojo = baja disponibilidad.

## 4. Recomendaciones para el Diagnóstico y Tratamiento para Pacientes con Atrofia Muscular Espinal

### 4.1 Recomendaciones para el Proceso Diagnóstico de la AME

El diagnóstico de AME-5q debe basarse en la confirmación genética de variantes patogénicas bialélicas en el gen *SMN1*, siendo la deleción homocigota del exón 7 la alteración más frecuente y clínicamente relevante, presente en aproximadamente el 95% de los pacientes, independientemente de la severidad fenotípica [1,32]. En pacientes con un fenotipo típico, especialmente en presentaciones de inicio temprano, el diagnóstico suele confirmarse mediante estudios moleculares sin requerir evaluaciones adicionales. En este contexto, el panel recomienda como estudio inicial el uso de MLPA o PCR cuantitativa para la detección de la deleción del exón 7 de *SMN1,* reservando la secuenciación de *SMN1* para casos con deleción heterocigota o hallazgos genéticos atípicos, con el objetivo de identificar variantes puntuales no detectables por métodos basados en dosificación génica [1,32,33] (Tabla 2). Asimismo, se recomienda la determinación del número de copias del gen *SMN2* como parte integral del algoritmo diagnóstico. Si bien no constituye un criterio diagnóstico *per se*, se trata de un marcador con importante valor pronóstico, dado que se correlaciona de manera inversa con la severidad clínica y contribuye a anticipar el curso clínico, incluso en etapas presintomáticas [1,7,33]. Finalmente, el panel recomienda avanzar en la implementación del cribado, tamizaje o pesquisa neonatal para AME, estrategia respaldada por algoritmos de manejo de lactantes diagnosticados por cribado neonatal y por experiencias piloto regionales, como la reportada en Brasil [20,28,34], así como por programas poblacionales consolidados, como el desarrollado en Alemania, que han demostrado que el diagnóstico y tratamiento presintomático se asocian a una probabilidad significativamente mayor de adquisición de hitos motores mayores, incluida la deambulación independiente, en comparación con cohortes diagnosticadas de forma sintomática [15,35]. La detección presintomática permite favorecer el inicio temprano de terapias modificadoras de la enfermedad, con impacto clínico relevante, en concordancia con estándares internacionales de cuidado [1,15,35] (Tabla 2).

**Tabla 2.** **Recomendaciones para el proceso diagnóstico de la AME**.

**Para el diagnóstico y la detección precoz de la AME se recomienda**
(a) Confirmar el diagnóstico mediante la demostración de variantes patogénicas bialélicas en el gen *SMN1*.
(b) Incluir como estudio inicial de confirmación diagnóstica la búsqueda de la deleción del exón 7 del gen *SMN1* mediante técnicas de MLPA o PCR cuantitativa.
(c) Determinar el número de copias del gen *SMN2* como parte del proceso diagnóstico.
(d) Realizar la secuenciación del gen *SMN1* en los casos en que se demuestre una sola deleción o se obtengan hallazgos genéticos atípicos, con el objetivo de descartar variantes puntuales.
(e) Considerar otras enfermedades neuromusculares y ampliar los estudios complementarios según las características de cada caso en pacientes con presentación clínica atípica o sin confirmación genética.
(f) Implementar el tamizaje neonatal para AME con el fin de favorecer la detección precoz de la enfermedad.

### 4.2 Recomendaciones para el Tratamiento Multidisciplinario 

En esta instancia se estratificaron a los pacientes en el grupo que no ha alcanzado la sedestación, que alcanzó la sedestación y el grupo de pacientes que caminan para facilitar las recomendaciones.

#### 4.2.1 Paciente con AME que no Ha Alcanzado la Sedestación

En los pacientes con AME que no han alcanzado la sedestación, el panel recomienda interpretar la respuesta al tratamiento de forma amplia, considerando tanto la mejoría como la estabilidad clínica como resultados relevantes (Tabla 3.1). Esta aproximación se basa en la historia natural progresiva de este subgrupo, en la cual la ausencia de deterioro constituye un efecto terapéutico clínicamente significativo [1,27,36].

**Tabla 3.1.** **Recomendaciones en paciente con AME que no ha alcanzado la sedestación**.

**Recomendaciones para el tratamiento respiratorio**	**Recomendaciones para el tratamiento deglutorio**	**Recomendaciones para el tratamiento de rehabilitación**
(a) Evaluar la función respiratoria mediante el examen físico, incluyendo frecuencia y amplitud respiratoria, eficacia de la tos y otros aspectos funcionales.	(a) Evaluar la función deglutoria mediante el examen físico, incluyendo succión, eficacia masticatoria, presencia de signos clínicos de aspiración y otros aspectos funcionales.	(a) Evaluar clínicamente el control postural, la presencia y el grado de escoliosis y el rango de movimiento articular con el fin de orientar la estrategia de rehabilitación.
(b) Realizar una evaluación de la función respiratoria como método de tamizaje mediante oximetría y capnografía transcutánea.	(b) Indicar una evaluación instrumental de la deglución mediante videofluoroscopia de deglución ante la presencia de signos clínicos de trastornos deglutorios.	(b) Evaluar la presencia de deformidades ortopédicas, incluyendo deformidades de los pies, retracción del tendón de Aquiles, cifosis y luxación de caderas.
(c) Indicar un estudio de sueño (polisomnografía) ante la sospecha clínica de hipoventilación.	(c) Evaluar la posible presencia de reflujo gastroesofágico.	(c) Valorar la magnitud de la debilidad muscular mediante el uso de escalas funcionales validadas, como CHOP INTEND.
(d) Implementar estrategias de higiene y soporte respiratorio, que incluyan aspiración oronasal, rehabilitación respiratoria/fisioterapia y técnicas de tos asistida, entre otras.		(d) Evaluar el desarrollo motor mediante escalas estandarizadas, como HINE.
(e) Indicar ventilación no invasiva ante la presencia de hipoventilación clínicamente manifiesta y confirmada por estudios complementarios.		(e) Indicar el uso diario de sistemas de sedestación, soportes posturales y dispositivos de posicionamiento en pacientes con trastornos posturales, con el objetivo de favorecer la alineación y corrección postural.
		(f) Indicar el uso diario de órtesis para miembros en pacientes con contracturas, con el fin de favorecer el estiramiento, la función y el mantenimiento del rango articular.
		(g) Recomendar que las órtesis, cuando estén indicadas, se utilicen durante el mayor tiempo que el paciente tolere, a fin de optimizar la efectividad de la intervención y la funcionalidad.
		(h) Indicar la realización de estiramientos una o dos veces al día para mejorar el rango articular.
		(i) Recomendar que la frecuencia mínima de los estiramientos sea de al menos 5 a 7 veces por semana para lograr un efecto terapéutico adecuado.
		(j) Recomendar que, en los pacientes que utilicen órtesis, la frecuencia mínima de uso sea de al menos 5 veces por semana para asegurar la efectividad de la intervención.
**Recomendaciones para terapias modificadoras de la enfermedad**		
(a) Nusinersen ha demostrado ser eficaz y seguro para el tratamiento de la enfermedad. Se recomienda evaluar su uso por parte del equipo tratante con el objetivo de modificar el curso de la enfermedad.		
(b) Risdiplam ha demostrado ser eficaz y seguro para el tratamiento de la enfermedad. Se recomienda evaluar su uso por parte del equipo tratante con el objetivo de modificar el curso de la enfermedad.		
(c) Onasemnogene abeparvovec ha demostrado ser eficaz y seguro para el tratamiento de la enfermedad en pacientes menores de 2 años. Se recomienda evaluar su uso por parte del equipo tratante con el objetivo de modificar el curso de la enfermedad.		

Las afirmaciones consensuadas subrayan la necesidad de evaluar la respuesta mediante escalas funcionales objetivas adaptadas a pacientes no sedentes, capaces de detectar cambios sutiles y reproducibles. En este contexto, la evaluación no debe limitarse a la adquisición de hitos motores mayores, sino centrarse en la evolución funcional global y en cambios pequeños pero sostenidos con relevancia clínica [1,37].

Asimismo, se recomienda incorporar parámetros respiratorios y bulbares como criterios válidos de respuesta, dado que la estabilidad o mejoría respiratoria, la reducción de complicaciones infecciosas y la preservación de la alimentación oral constituyen indicadores de alto valor clínico, en concordancia con la Tabla 3.1 [1,22].

Respecto a la frecuencia de evaluación, se acordó que los intervalos deben ser flexibles y ajustarse a la situación clínica individual, equilibrando el seguimiento con la factibilidad de implementación en contextos reales de atención en la región [1,22,27].

Finalmente, se alcanzó consenso en mantener la TME aun en ausencia de mejoría objetivable a corto plazo, considerando que la estabilidad clínica sostenida en los dominios funcionales, respiratorios y bulbares definidos constituye un argumento válido para su continuidad [1,22,27] (Tabla 3.1).

#### 4.2.2 Paciente con AME que Ha Alcanzado la Sedestación 

En los pacientes con AME que han alcanzado la sedestación, el panel alcanzó consenso en que la evaluación de la respuesta a la TME debe adoptar un enfoque funcional integral (Tabla 3.2). Este subgrupo presenta un perfil clínico intermedio, con posibilidad de estabilidad prolongada y, en algunos casos, mejoría funcional parcial [1,2,22]. Las afirmaciones consensuadas destacan la utilidad de escalas motoras objetivas apropiadas para pacientes sedestadores, que permiten cuantificar cambios clínicamente relevantes en la función axial, proximal y de miembros superiores. En este contexto, la respuesta no debe interpretarse sólo como adquisición de nuevos hitos motores, sino también como preservación de capacidades previamente alcanzadas, aspecto particularmente relevante en una enfermedad progresiva [1,37,38]. Asimismo, se recomienda la inclusión de parámetros respiratorios, considerando clínicamente significativa la estabilidad funcional, la ausencia de progresión en el soporte ventilatorio y la reducción de eventos respiratorios agudos, en línea con los dominios evaluados [1,22]. El panel también recomienda incorporar criterios bulbares y nutricionales, como la estabilidad en la alimentación oral y la disminución de complicaciones asociadas, dada su relevancia sobre la calidad de vida y la evolución global en este grupo [1,22]. En relación con la periodicidad, se recomienda una frecuencia de evaluación flexible y ajustada al estado clínico y a la disponibilidad de recursos, buscando una implementación realista sin comprometer la detección de cambios relevantes [1,22]. Finalmente, se considera apropiado mantener la TME aun sin mejoría motora objetivable a corto plazo, si existe estabilidad clínica sostenida en los dominios consensuados [1,22,27] (Tabla 3.2).

**Tabla 3.2.** **Recomendaciones en paciente con AME que ha alcanzado la sedestación**.

**Recomendaciones para el tratamiento respiratorio**	**Recomendaciones para el tratamiento deglutorio**	**Recomendaciones para el tratamiento de rehabilitación**
(a) Evaluar la función respiratoria mediante el examen físico, incluyendo frecuencia y amplitud respiratoria, eficacia de la tos y otros aspectos funcionales.	(a) Evaluar la función deglutoria mediante el examen físico, incluyendo succión, eficacia masticatoria, presencia de signos clínicos de aspiración y otros aspectos funcionales.	(a) Evaluar clínicamente el control postural, la presencia y el grado de escoliosis y el rango de movimiento articular, con el fin de diseñar o adaptar un programa de rehabilitación acorde.
(b) Realizar una evaluación de la función respiratoria como método de tamizaje mediante oximetría.	(b) Indicar una evaluación instrumental de la deglución mediante videofluoroscopia de deglución ante la presencia de signos clínicos de trastornos deglutorios.	(b) Evaluar la presencia de deformidades ortopédicas, incluyendo deformidades de los pies y luxación de caderas, que puedan requerir tratamiento específico.
(c) Indicar la evaluación de la función respiratoria mediante espirometría con medición del flujo pico de tos (PCF, Peak Cough Flow), siempre que la edad y el grado de cooperación del paciente lo permitan.		(c) Utilizar la escala CHOP INTEND para evaluar el grado de debilidad en pacientes ≤24 meses con debilidad significativa.
(d) Indicar un estudio de sueño (polisomnografía) ante la sospecha clínica de hipoventilación.		(d) Evaluar la presencia y el grado de debilidad muscular mediante escalas funcionales validadas, como la EK2.
(e) Evaluar la posible presencia de reflujo gastroesofágico.		(e) Aplicar otras escalas funcionales durante el seguimiento, tales como HFMSE, RULM y MFM, con el objetivo de evaluar cambios en la función motora.
(f) Indicar ventilación no invasiva ante la presencia de hipoventilación clínicamente manifiesta y confirmada por estudios complementarios.		(f) Indicar el uso diario de sistemas de sedestación, soportes posturales y dispositivos de posicionamiento en pacientes con trastornos posturales, con el objetivo de favorecer la alineación y corrección postural.
		(g) Indicar el uso diario de órtesis para miembros en pacientes con contracturas, con el fin de favorecer el estiramiento, la función y el mantenimiento del rango articular.
		(h) Recomendar que las órtesis, cuando estén indicadas, se utilicen durante el mayor tiempo que el paciente tolere, a fin de optimizar la efectividad de la intervención.
		(i) Recomendar que la frecuencia mínima de los estiramientos sea de al menos 5 a 7 veces por semana para mejorar el rango articular.
		(j) Recomendar que, en los pacientes que utilicen órtesis, la frecuencia mínima de uso sea de al menos 5 veces por semana para asegurar la efectividad de la intervención.
**Recomendaciones para terapias modificadoras de la enfermedad**		
(a) Nusinersen ha demostrado ser eficaz y seguro para el tratamiento de la enfermedad. Se recomienda evaluar su uso por parte del equipo tratante con el objetivo de modificar el curso de la enfermedad.		
(b) Risdiplam ha demostrado ser eficaz y seguro para el tratamiento de la enfermedad. Se recomienda evaluar su uso por parte del equipo tratante con el objetivo de modificar el curso de la enfermedad.		
(c) Onasemnogene abeparvovec ha demostrado ser eficaz y seguro para el tratamiento de la enfermedad en pacientes menores de 2 años. Se recomienda evaluar su uso por parte del equipo tratante con el objetivo de modificar el curso de la enfermedad.		

#### 4.2.3 Paciente con AME que Caminan

En los pacientes con AME que han alcanzado la deambulación, el panel alcanzó consenso en que la evaluación de la respuesta a la TME debe priorizar la preservación de la capacidad de marcha y la función motora ambulatoria, ya que su pérdida constituye un hito clínico crítico con impacto funcional y pronóstico [1,2,22,27] (Tabla 3.3). En este subgrupo, la respuesta terapéutica suele expresarse como enlentecimiento o estabilidad del deterioro más que como ganancia motora evidente; por ello, el uso de escalas y pruebas funcionales sensibles para marcha, resistencia y desempeño motor resulta clave para detectar cambios clínicamente relevantes [1,22,39]. El panel destaca además la importancia del seguimiento longitudinal durante la adolescencia y la adultez temprana, periodos en los que puede observarse declinación funcional aun en pacientes clínicamente estables [1,22]. Asimismo, se recomienda mantener una evaluación sistemática de la función respiratoria, considerando que el compromiso puede desarrollarse de forma insidiosa y coexistir con una marcha aparentemente conservada [1,22].

**Tabla 3.3.** **Recomendaciones en paciente con AME que camina**.

**Recomendaciones para el tratamiento respiratorio**	**Recomendaciones para el tratamiento deglutorio**	**Recomendaciones para el tratamiento de rehabilitación**
(a) Ser evaluados mediante el examen físico respecto a la función respiratoria (frecuencia, amplitud, eficacia de la tos y otros aspectos funcionales).	(a) Ser evaluados mediante el examen físico respecto a la función deglutoria (succión, eficacia masticatoria, presencia de signos clínicos de aspiración y otros aspectos funcionales).	(a) Evaluar clínicamente el control postural, la presencia y el grado de escoliosis y el rango de movimiento articular, con el fin de diseñar o adaptar un programa de rehabilitación acorde a sus necesidades.
(b) Ser evaluados mediante espirometría, siempre que la edad y el grado de cooperación lo permitan.	(b) Ser evaluados mediante videofluoroscopía de deglución ante la presencia de signos clínicos de trastornos de la deglución.	(b) Utilizar durante el seguimiento escalas funcionales como HFMSE, RULM y pruebas funcionales cronometradas (Timed Function Tests, TFTs) para evaluar cambios en el estado funcional.
(c) Ser evaluados mediante estudio de sueño (polisomnografía) ante la sospecha clínica de hipoventilación.	(c) Ser evaluados nutricionalmente por especialistas, de forma periódica, para valorar peso, talla, ingesta calórica e identificar signos de malnutrición.	(c) Identificar la presencia de caídas durante la deambulación para su abordaje específico en el plan de rehabilitación.
		(d) Indicar el uso diario de órtesis en miembros en presencia de contracturas, con el objetivo de favorecer el estiramiento, la función y el mantenimiento del rango articular.
**Recomendaciones para terapias modificadoras de la enfermedad**		
(a) Nusinersen ha demostrado ser eficaz y seguro para el tratamiento de la enfermedad. Se recomienda evaluar su uso por parte del equipo tratante con el objetivo de modificar el curso de la enfermedad.		
(b) Risdiplam ha demostrado ser eficaz y seguro para el tratamiento de la enfermedad. Se recomienda evaluar su uso por parte del equipo tratante con el objetivo de modificar el curso de la enfermedad.		
(c) Onasemnogene abeparvovec ha demostrado ser eficaz y seguro para el tratamiento de la enfermedad en pacientes menores de 2 años. Se recomienda evaluar su uso por parte del equipo tratante con el objetivo de modificar el curso de la enfermedad.		
**Recomendaciones para terapias modificadoras de la enfermedad en pacientes mayores de 18 años**		
(a) Nusinersen ha demostrado ser eficaz y seguro para el tratamiento de la enfermedad.		
(b) Se recomienda evaluar el uso de nusinersen por parte del equipo tratante, con el objetivo de modificar el curso de la enfermedad.		
(c) Risdiplam ha demostrado ser eficaz para el tratamiento de la enfermedad.		
(d) Se recomienda evaluar el uso de risdiplam por parte del equipo tratante, con el objetivo de modificar el curso de la enfermedad.		

En adultos con AME (≥18 años), se aconseja un enfoque multidimensional orientado a preservar la función y prevenir la pérdida de habilidades, reconociendo que la respuesta puede manifestarse principalmente como estabilidad clínica sostenida. En ausencia de mejoría o estabilización, se considera apropiado definir la continuidad del tratamiento modificador sobre la base de un análisis clínico integral, que contemple objetivos individualizados del paciente con impacto en las actividades de la vida diaria, incluyendo parámetros respiratorios, bulbares y la percepción de la enfermedad [1,22,26,39] . En este contexto, la estabilidad funcional sostenida constituye un resultado clínicamente relevante, particularmente cuando las herramientas disponibles pueden no ser suficientemente sensibles para detectar cambios significativos [22,27,39,40] (Tablas 3.3 y 3.4).

**Tabla 3.4.** **Recomendaciones transversales comunes a todos los estados funcionales**.

**Recomendaciones**	
**Seguimiento multidisciplinario**	• Realizar un seguimiento clínico integral por un equipo multidisciplinario con experiencia en AME.
	• Ajustar la frecuencia de las evaluaciones según la edad y el estado funcional del paciente, de acuerdo con el criterio del equipo tratante.
	• Garantizar la evaluación periódica por especialistas según compromiso orgánico.
**Frecuencia mínima de seguimiento**	• Realizar evaluaciones periódicas con una frecuencia mínima de cada 3 a 6 meses.
**Capacitación de cuidadores**	• Proveer entrenamiento a los cuidadores primarios para el manejo respiratorio, nutricional y de rehabilitación según corresponda.

### 4.3 Recomendaciones sobre la Respuesta al Tratamiento

La evaluación de la respuesta a la TME en la AME requiere un enfoque integral que contemple la heterogeneidad clínica, funcional y evolutiva de los pacientes. El panel recomienda que la respuesta no debe interpretarse de manera aislada ni transversal, sino en un marco longitudinal, individualizado y considerando diferentes dominios o dimensiones de la enfermedad, acorde con la evidencia de ensayos clínicos, datos de vida real y consensos internacionales [1,8,22,26,27] (Tabla 4). Se reconoce que la respuesta terapéutica está condicionada por el estado basal del paciente, incluyendo el fenotipo clínico, la edad de inicio de los síntomas, el tiempo transcurrido hasta el inicio del tratamiento y el nivel funcional al momento de su instauración, particularmente en relación con la adquisición de hitos motores como la sedestación o la deambulación. Asimismo, influyen variables contextuales y ortopédicas, como la presencia de escoliosis, la realización de cirugías correctivas y el acceso oportuno al tratamiento. Estos factores determinan el potencial de mejoría o estabilización y justifican evitar criterios uniformes no contextualizados [8,22,41,42]. El panel destaca que la evaluación motora sigue siendo central, pero debe interpretarse según la historia natural; en determinados pacientes, la estabilidad funcional sostenida constituye un resultado clínicamente relevante. Asimismo, se recomienda integrar de forma sistemática dominios no motores (respiratorio, bulbar/nutricional), calidad de vida y reducción/estabilidad de hospitalizaciones relacionadas con la enfermedad, dado su impacto en morbimortalidad y en actividades de la vida diaria [22,42]. Para objetivar la respuesta, se recomienda utilizar herramientas validadas seleccionadas por edad, nivel funcional y dominio clínico, incluyendo CHOP-INTEND en menores de 24 meses, HFMSE y RULM en no ambulatorios, y HFMSE junto con 6MWT en ambulatorios y cuestionarios de calidad de vida (PedsQL neuromuscular). En adultos, se remarca la utilidad y limitaciones de escalas motoras y funcionales en el seguimiento longitudinal [22,37,38,43]. La calidad de vida debe ser evaluada en todos los grupos etarios y niveles de función motora, utilizando herramientas validadas y adaptadas a la edad y al perfil del paciente [44]. Respecto a la frecuencia de evaluación, se propone que la decisión de continuidad o interrupción se base en el cumplimiento de objetivos terapéuticos, con intervalos orientativos de 12–18 meses en inicios precoces y 18–24 meses en inicios tardíos, individualizando según edad, grado funcional y tiempo de evolución. En cuanto al inicio de terapias modificadoras de la enfermedad, se recomienda comenzarlo ante la presencia de síntomas y alteración funcional objetivable mediante escalas motoras y/o funcionales, incluyendo también a los pacientes adultos cuando exista compromiso clínico documentable [8,9,10,11,12,13,14,15,16,17,18,19]. En contextos donde no se dispone de pesquisa neonatal, se enfatiza que el inicio del tratamiento debe considerarse desde la aparición de los primeros síntomas, dado que la indicación y el diagnóstico son fundamentalmente clínicos, siendo las escalas herramientas útiles para el seguimiento evolutivo [45,46]. El panel recomienda iniciar la TME lo más rápidamente posible, considerando el impacto del tiempo de evolución sobre el potencial de respuesta [8,9,10,11,12,13,14,15,16,17,18,19,27]. Finalmente, se determina que la interrupción de la TME debe considerarse de manera individualizada, dentro de un marco clínico y ético, particularmente ante falta de beneficio clínicamente relevante, aparición de eventos adversos graves atribuibles al fármaco o enfermedad avanzada con expectativas mínimas de cambio (por ejemplo, dependencia ventilatoria permanente y ausencia de movimientos voluntarios), tanto en población pediátrica como adulta [27,28,47] (Tabla 4).

**Tabla 4.** **Recomendaciones sobre la respuesta a la terapia modificadora de la enfermedad (TME)**.

**I-Determinantes de la respuesta a la TME**	**II-Objetivos de respuesta a la TME**
En todos los pacientes con AME, para fijar los objetivos de respuesta a la TME se recomienda considerar los siguientes determinantes clínicos y contextuales:	En todos los pacientes con AME, se recomienda que la evaluación de la respuesta a la TME contemple los siguientes objetivos clínicos y centrados en el paciente:
(a) La funcionalidad basal, incluyendo la capacidad de alcanzar la sedestación y la deambulación.	**(a) Función respiratoria, deglutoria y bulbar**.
(b) El tipo de AME (tipos I, II, III y IV).	- Dentro de cada una de estas evaluaciones, se recomienda considerar la mejoría como parámetro de respuesta al tratamiento.
(c) La edad del paciente al momento de iniciar el tratamiento.	- En determinados pacientes, la estabilidad de cada una de estas funciones puede considerarse un parámetro adecuado de respuesta, en función de los determinantes de respuesta, tales como el tipo de AME, la edad, el tiempo de evolución y el nivel funcional basal.
(d) El tiempo de evolución de la enfermedad.	**(b) La calidad de vida**.
(e) La presencia de escoliosis y, de corresponder, la realización de cirugía correctiva.	- Dentro de la evaluación de la calidad de vida, se recomienda considerar la mejoría como parámetro de respuesta al tratamiento.
(f) El acceso al tratamiento.	**(c) Número de ingresos hospitalarios/año relacionados con la enfermedad**
	- Dentro de la evaluación de los ingresos hospitalarios/año, se recomienda considerar la reducción de estos como parámetro de respuesta al tratamiento.
	- En determinados pacientes, la estabilidad en la cantidad de ingresos hospitalarios/año puede considerarse un parámetro adecuado de respuesta, en función de los determinantes de respuesta.
**III-Herramientas de evaluación de respuesta a la TME**	
En los pacientes con atrofia muscular espinal (AME), se recomienda en la evaluación de la respuesta a la TME, utilizar herramientas validadas, seleccionadas según la edad, el nivel funcional y el dominio clínico a evaluar:	
**Función motora**	
(a) La escala CHOP-INTEND para evaluar la función motora en pacientes ≤24 meses.	
(b) Las escalas HFMSE y RULM para evaluar la función motora en pacientes mayores de 24 meses que no caminan.	
(c) Las escalas HFMSE y la prueba de la marcha de 6 minutos (6MWT) para evaluar la función motora en pacientes mayores de 24 meses que caminan.	
(d) La escala EK2 para evaluar la función motora en pacientes mayores de 4 años que no caminan.	
**Calidad de vida**	
(a) El cuestionario PedsQL – módulo neuromuscular, versión respondida por el cuidador, para evaluar la calidad de vida en pacientes entre 2 y 18 años.	
(b) El cuestionario PedsQL – módulo neuromuscular, versión respondida por el paciente, para evaluar la calidad de vida en pacientes entre 5 y 18 años.	
**Función respiratoria**	
(a) La espirometría con evaluación de la capacidad vital forzada (CVF), el volumen espiratorio forzado en el primer segundo (VEF1) y el cociente VEF1/CVF en pacientes capaces de realizar la prueba de forma confiable (generalmente ≥5 años).	
(b) Oximetría/capnografía en pacientes más jóvenes o en aquellos que no puedan colaborar adecuadamente con la espirometría.	
(c) La capnografía transcutánea para evaluar la función respiratoria en pacientes menores de 4 años.	
**Función bulbar**	
(a) La videofluoroscopia de deglución para evaluar la función bulbar en pacientes con AME.	
(b) La herramienta OrSAT (Oral and Swallowing Abilities Tool) para evaluar la función bulbar en pacientes menores de 2 años.	
(c) Herramientas estructuradas de evaluación bulbar en pacientes mayores de 2 años, como la escala SAFE (Swallowing Ability and Function Evaluation), si bien con disponibilidad limitada de versiones validadas al español.	
(d) La escala EK2 (ítems bulbares) para evaluar la función bulbar en pacientes mayores de 2 años.	
**IV-Frecuencia de evaluación de respuesta a la TME**	**V-Inicio e interrupción de la TME**
Se recomienda que la evaluación de la respuesta a la TME y la decisión sobre la continuidad o interrupción de este se realicen en función del cumplimiento de los objetivos terapéuticos definidos, considerando los siguientes intervalos:	Se recomienda **iniciar** la TME en presencia de síntomas y alteración funcional objetivada mediante escalas motoras y/o funcionales.
(a) Cada 12 a 18 meses en pacientes que inician el tratamiento de forma precoz.	(a) terapia modificadora de la enfermedad (TME) lo antes posible después de la confirmación diagnóstica de AME, incluidos los pacientes presintomáticos identificados mediante tamizaje neonatal. En pacientes sintomáticos, los síntomas clínicos y el deterioro funcional objetivamente medible deben documentarse como parte de la evaluación basal.
(b) Cada 18 a 24 meses en pacientes que inician el tratamiento de forma tardía.	(b) En los casos en los que esté indicado, se recomienda iniciar la TME lo más rápidamente posible.
(c) La frecuencia de las evaluaciones debe individualizarse según las características de cada paciente, particularmente el grado funcional, la edad y el tiempo de evolución de la enfermedad.	**La interrupción** de la TME debe ser considerada de manera individualizada, en el contexto de la evaluación clínica y ética, en las siguientes situaciones:
	(a) En pacientes pediátricos, cuando el equipo tratante y el comité de ética consideren que existe falta de beneficio clínicamente relevante.
	(b) En pacientes pediátricos, ante la presencia de eventos adversos graves asociados al medicamento.
	(c) En pacientes pediátricos con enfermedad en estadios avanzados cuando existan mínimas o nulas expectativas de cambio con el tratamiento.
	(d) En pacientes adultos, cuando el equipo tratante considere que existe falta de beneficio clínicamente relevante.
	(e) En pacientes adultos, ante la presencia de eventos adversos graves asociados al medicamento.
	(f) En pacientes adultos con enfermedad en estadios avanzados (dependencia ventilatoria permanente, ausencia de movimientos voluntarios).

## 5. Discusión 

Este consenso latinoamericano sintetiza recomendaciones para el diagnóstico, el abordaje multidisciplinario y la evaluación de la respuesta a la TME en AME, integrando la evidencia disponible con la experiencia regional y con información contextual sobre acceso real en LATAM (Tabla 2, Tablas 3.1,3.2,3.3,3.4 y Tabla 4, y Fig. 3). El cambio de paradigma generado por la TME ha transformado de manera sustancial la historia natural de la atrofia muscular espinal (AME), dando lugar a nuevas trayectorias clínicas y a la emergencia de fenotipos previamente no descritos, configurando a la enfermedad como una condición crónica con necesidad de seguimiento longitudinal y objetivos terapéuticos individualizados, particularmente cuando el tratamiento se inicia de manera precoz [8,9,10,11,12,13,14,15,16,17,18,19]. En los pacientes tratados de forma temprana se ha observado la adquisición de hitos motores previamente impensables, lo que ha generado nuevos desafíos para los sistemas de salud, los equipos médicos especializados, así como para los pacientes y sus familias, exigiendo una adaptación dinámica de los modelos de atención y de los cuidados multidisciplinarios a estos nuevos perfiles clínicos [8,12,13,14,48] Recientemente, se han reportado nuevas estrategias terapéuticas y esquemas de dosificación en evaluación o aprobación en algunos contextos regulatorios, lo que refleja la evolución continua del tratamiento de la AME [49,50,51,52]. En este contexto, el seguimiento longitudinal de las personas con AME no solo debe enfocarse en la prevención y el manejo de las complicaciones asociadas a la enfermedad, sino que también requiere la incorporación sistemática de herramientas estandarizadas que permitan una evaluación objetiva, reproducible y comparable de la respuesta terapéutica [1,22,27,47]. En este escenario, resulta crítico mantener la alineación con estándares internacionales de cuidado, adaptando su implementación a las asimetrías de recursos y acceso características de la región [1,22,27,28,47].

Un hallazgo relevante de la encuesta regional fue la marcada heterogeneidad en los componentes clave del proceso asistencial (Tabla 1). Si bien la mayoría de los encuestados reportó disponibilidad de MLPA, solo el 35% indicó que este estudio se encuentra incluido dentro del sistema público de salud, mientras que el resto depende de financiamiento privado o del apoyo de la industria. Asimismo, se reportó acceso a al menos una terapia modificadora; sin embargo, persisten brechas en los tiempos de confirmación genética, la ausencia de pesquisa neonatal en la mayoría de los países y demoras frecuentes entre el diagnóstico y el inicio del tratamiento, además de acceso incompleto a unidades multidisciplinarias y a programas de ventilación domiciliaria. Estos resultados refuerzan que la efectividad de la TME depende, en la práctica, de un sistema que permita diagnóstico oportuno, derivación temprana y soporte multidisciplinario continuo [22,42,45,46,53].

En línea con la literatura, el consenso enfatiza que la respuesta a la TME no debe basarse exclusivamente en la ganancia de hitos motores, sino en una evaluación por dominios y centrada en el paciente, considerando mejoría o estabilidad según determinantes basales como tipo de AME, edad al inicio, nivel funcional y tiempo de evolución [1,22,27]. Esta aproximación resulta particularmente pertinente en pacientes con mayor compromiso motor, en quienes la estabilización clínica puede representar un beneficio clínicamente significativo frente a una historia natural progresiva, y se alinea con la evidencia de ensayos pivotales que muestran magnitudes de respuesta distintas según el momento de inicio y el estadio clínico [8,10,11,12,13,48].

La estratificación por nivel funcional (no sedestadores, sedestadores no ambulatorios y ambulatorios) aporta una estructura práctica para operacionalizar objetivos realistas y comparables. En no sedestadores, integrar dominios respiratorios y bulbares en la definición de respuesta es esencial, dado el impacto del compromiso respiratorio y deglutorio sobre morbimortalidad, hospitalizaciones y calidad de vida [1,22,27]. En sedestadores, la preservación de capacidades previamente alcanzadas, en particular la funcionalidad de las extremidades superiores y el seguimiento sistemático de función respiratoria, bulbar/nutricional y calidad de vida son componentes críticos del beneficio terapéutico, más allá de la adquisición de nuevos hitos motores [1,22,27]. En pacientes ambulatorios, la desaceleración del deterioro, la evaluación de resistencia y desempeño, y la detección de caídas y fatiga como señales de progresión funcional permiten ajustar rehabilitación y seguimiento de manera más sensible, en especial durante adolescencia y adultez temprana [1,22,27].

La recomendación de utilizar herramientas validadas según edad, dominio y estado funcional apunta a reducir la variabilidad clínica y facilitar decisiones reproducibles. La inclusión de CHOP-INTEND en menores de 24 meses, HFMSE/RULM en no ambulatorios, 6MWT y HFMSE en ambulatorios es coherente con su uso extendido en investigación clínica y práctica, y favorece la detección de cambios sutiles clínicamente relevantes cuando el objetivo principal es la estabilización o el enlentecimiento de la progresión [37,38,43,47,54,55]. Asimismo, el énfasis en instrumentos para medir la función bulbar y en métricas respiratorias objetivas contribuye a una evaluación más completa del impacto terapéutico [22,47].

Respecto a la pesquisa neonatal, su implementación emerge como prioridad sanitaria regional. Las experiencias piloto y algoritmos de manejo de lactantes detectados por cribado respaldan su factibilidad y su potencial para maximizar el beneficio de las terapias al permitir tratamiento presintomático o muy precoz lo que puede determinar la posibilidad de que un paciente sea capaz de caminar o no [45,46]. En paralelo, el consenso propone un marco pragmático para la toma de decisiones sobre continuidad e interrupción de la TME, incorporando criterios clínicos y éticos (beneficio clínicamente relevante, eventos adversos graves, enfermedad avanzada), lo que resulta especialmente útil en entornos con comités de elegibilidad y con restricciones de acceso [1,22,26,53,56].

Este trabajo presenta limitaciones. La encuesta describe percepciones de profesionales y puede estar sujeta a sesgo de selección, sobrerrepresentación de ámbitos urbanos y variaciones en el conocimiento de circuitos administrativos locales. Además, el método de consenso, aunque estructurado, depende de la composición del panel y de la evidencia disponible; en áreas con datos limitados, algunas recomendaciones reflejan la mejor síntesis posible entre evidencia y aplicabilidad regional. Adicionalmente, si bien el apoyo psicológico al paciente y su familia, así como el asesoramiento genético, constituyen elementos fundamentales en la atención integral de la atrofia muscular espinal (AME), estos no fueron abordados de forma específica en el presente consenso, dado que no son exclusivos de esta enfermedad y deberían integrarse de manera estructurada en los programas de atención multidisciplinaria para enfermedades neuromusculares en general. Por otra parte, la ausencia de centros de referencia adecuadamente estructurados en varios de los sistemas de salud de la región, junto con la heterogeneidad organizativa y de recursos entre países, representa una limitación relevante que puede condicionar la interpretación y generalización de los resultados. Por otro lado el acceso a las terapias modificadoras de la enfermedad puede diferir entre pacientes pediátricos y adultos en los distintos sistemas de salud de Latinoamérica, y la inclusión de participantes dedicados exclusivamente a la atención de pacientes adultos podría haber contribuido a la heterogeneidad observada en las respuestas relacionadas con el acceso al tratamiento [25,42,46,53]. Sin embargo, la fortaleza del documento radica en su enfoque práctico, su alineación con estándares internacionales y la integración explícita de barreras de implementación en LATAM.

Finalmente, los resultados señalan líneas prioritarias para investigación e implementación: expansión de pesquisa neonatal, reducción de demoras diagnósticas y terapéuticas, fortalecimiento de redes multidisciplinarias, estandarización de métricas por dominios y generación de datos regionales prospectivos (registros) que permitan evaluar efectividad, seguridad, costos indirectos y desenlaces centrados en el paciente en escenarios reales de atención [26,46,47,53].

En conclusión, el presente consenso latinoamericano propone recomendaciones prácticas para el diagnóstico, el manejo multidisciplinario y la evaluación de respuesta a la TME en AME, adaptadas al contexto de LATAM y alineadas con la evidencia y estándares internacionales. Las recomendaciones enfatizan una evaluación longitudinal por dominios (motor, respiratorio, bulbar/nutricional, calidad de vida y hospitalizaciones) y la estratificación por nivel funcional para definir objetivos realistas, incluyendo la estabilidad clínica como resultado relevante en subgrupos seleccionados. La encuesta regional evidencia brechas persistentes en pesquisa neonatal, tiempos de acceso y disponibilidad homogénea de recursos multidisciplinarios, subrayando la necesidad de estrategias regionales para mejorar diagnóstico oportuno, inicio temprano de la TME y seguimiento estandarizado.

En conjunto, este primer consenso latinoamericano constituye un hito inicial y un punto de partida para el desarrollo de una agenda colaborativa en AME en la región. El proceso ha permitido integrar la visión y experiencia de especialistas de 15 países, identificando de manera consensuada brechas prioritarias y oportunidades de mejora a lo largo del continuo de atención, desde el diagnóstico temprano hasta el seguimiento de la respuesta a la TME. A partir de este esfuerzo, se sientan las bases para la consolidación de un grupo de trabajo regional orientado a profundizar la estandarización de los cuidados multidisciplinarios, la implementación de métricas armonizadas y la generación de evidencia propia que respalde la toma de decisiones clínicas y sanitarias en LATAM.

## References

[1] Mercuri E, Finkel RS, Muntoni F, Wirth B, Montes J, Main M (2018). Diagnosis and management of spinal muscular atrophy: Part 1: Recommendations for diagnosis, rehabilitation, orthopedic and nutritional care. *Neuromuscular Disorders: NMD*.

[2] Annoussamy M, Seferian AM, Daron A, Péréon Y, Cances C, Vuillerot C (2021). Natural history of Type 2 and 3 spinal muscular atrophy: 2-year NatHis-SMA study. *Annals of Clinical and Translational Neurology*.

[3] Lunn MR, Wang CH (2008). Spinal muscular atrophy. *Lancet (London, England)*.

[4] Verhaart IEC, Robertson A, Wilson IJ, Aartsma-Rus A, Cameron S, Jones CC (2017). Prevalence, incidence and carrier frequency of 5q-linked spinal muscular atrophy - a literature review. *Orphanet Journal of Rare Diseases*.

[5] Prior TW, Leach ME, Finanger E *Spinal Muscular Atrophy. GeneReviews® [Internet]*.

[6] Calucho M, Bernal S, Alías L, March F, Venceslá A, Rodríguez-Álvarez FJ (2018). Correlation between SMA type and SMN2 copy number revisited: An analysis of 625 unrelated Spanish patients and a compilation of 2834 reported cases. *Neuromuscular Disorders: NMD*.

[7] Feldkötter M, Schwarzer V, Wirth R, Wienker TF, Wirth B (2002). Quantitative analyses of SMN1 and SMN2 based on real-time lightCycler PCR: fast and highly reliable carrier testing and prediction of severity of spinal muscular atrophy. *American Journal of Human Genetics*.

[8] Finkel RS, Mercuri E, Darras BT, Connolly AM, Kuntz NL, Kirschner J (2017). Nusinersen versus Sham Control in Infantile-Onset Spinal Muscular Atrophy. *The New England Journal of Medicine*.

[9] Mercuri E, Darras BT, Chiriboga CA, Day JW, Campbell C, Connolly AM (2018). Nusinersen versus Sham Control in Later-Onset Spinal Muscular Atrophy. *The New England Journal of Medicine*.

[10] Day JW, Finkel RS, Chiriboga CA, Connolly AM, Crawford TO, Darras BT (2021). Onasemnogene abeparvovec gene therapy for symptomatic infantile-onset spinal muscular atrophy in patients with two copies of SMN2 (STR1VE): an open-label, single-arm, multicentre, phase 3 trial. *The Lancet. Neurology*.

[11] Baranello G, Darras BT, Day JW, Deconinck N, Klein A, Masson R (2021). Risdiplam in Type 1 Spinal Muscular Atrophy. *The New England Journal of Medicine*.

[12] Masson R, Mazurkiewicz-Bełdzińska M, Rose K, Servais L, Xiong H, Zanoteli E (2022). Safety and efficacy of risdiplam in patients with type 1 spinal muscular atrophy (FIREFISH part 2): secondary analyses from an open-label trial. *The Lancet. Neurology*.

[13] Mercuri E, Deconinck N, Mazzone ES, Nascimento A, Oskoui M, Saito K (2022). Safety and efficacy of once-daily risdiplam in type 2 and non-ambulant type 3 spinal muscular atrophy (SUNFISH part 2): a phase 3, double-blind, randomised, placebo-controlled trial. *The Lancet. Neurology*.

[14] Finkel RS, Servais L, Vlodavets D, Zanoteli E, Mazurkiewicz-Bełdzińska M, Jong YJ (2025). Risdiplam in Presymptomatic Spinal Muscular Atrophy. *The New England Journal of Medicine*.

[15] De Vivo DC, Bertini E, Swoboda KJ, Hwu WL, Crawford TO, Finkel RS (2019). Nusinersen initiated in infants during the presymptomatic stage of spinal muscular atrophy: Interim efficacy and safety results from the Phase 2 NURTURE study. *Neuromuscular Disorders: NMD*.

[16] Maggi L, Bello L, Bonanno S, Govoni A, Caponnetto C, Passamano L (2020). Nusinersen safety and effects on motor function in adult spinal muscular atrophy type 2 and 3. *Journal of Neurology, Neurosurgery, and Psychiatry*.

[17] Matesanz SE, Curry C, Gross B, Rubin AI, Linn R, Yum SW (2020). Clinical Course in a Patient With Spinal Muscular Atrophy Type 0 Treated With Nusinersen and Onasemnogene Abeparvovec. *Journal of Child Neurology*.

[18] Mendell JR, Al-Zaidy S, Shell R, Arnold WD, Rodino-Klapac LR, Prior TW (2017). Single-Dose Gene-Replacement Therapy for Spinal Muscular Atrophy. *The New England Journal of Medicine*.

[19] Mendell JR, Al-Zaidy SA, Lehman KJ, McColly M, Lowes LP, Alfano LN (2021). Five-Year Extension Results of the Phase 1 START Trial of Onasemnogene Abeparvovec in Spinal Muscular Atrophy. *JAMA Neurology*.

[20] Glascock J, Sampson J, Haidet-Phillips A, Connolly A, Darras B, Day J (2018). Treatment Algorithm for Infants Diagnosed with Spinal Muscular Atrophy through Newborn Screening. *Journal of Neuromuscular Diseases*.

[21] Wang CH, Finkel RS, Bertini ES, Schroth M, Simonds A, Wong B (2007). Consensus statement for standard of care in spinal muscular atrophy. *Journal of Child Neurology*.

[22] Finkel RS, Mercuri E, Meyer OH, Simonds AK, Schroth MK, Graham RJ (2018). Diagnosis and management of spinal muscular atrophy: Part 2: Pulmonary and acute care; medications, supplements and immunizations; other organ systems; and ethics. *Neuromuscular Disorders: NMD*.

[23] Wohnrade C, Velling AK, Mix L, Wurster CD, Cordts I, Stolte B (2023). Health-Related Quality of Life in Spinal Muscular Atrophy Patients and Their Caregivers-A Prospective, Cross-Sectional, Multi-Center Analysis. *Brain Sciences*.

[24] Saenz V, Chlistalla M, Carlos N, Castiglioni C, Soledad Monges M, Servais L (2025). Patient and caregiver spinal muscular atrophy treatment attribute preferences in Latin America. *Journal of Neuromuscular Diseases*.

[25] Batista EC, Zanoteli E, Monfardini F, Santos GPD, Silva GS, Berwanger O (2024). Longitudinal data collection in pediatric and adult patients with 5q spinal muscular atrophy in Latin America: LATAM RegistrAME study - a clinical registry study protocol. *Einstein (Sao Paulo, Brazil)*.

[26] Ropars J, Grimaldi L, Quijano-Roy S (2025). Real-world evidence in spinal muscular atrophy: addressing heterogeneity of populations and treatment comparisons. *The Lancet Regional Health*.

[27] Pitarch Castellano I, Cabrera-Serrano M, Calvo Medina R, Cattinari MG, Espinosa García S, Fernández-Ramos JA (2022). Delphi consensus on recommendations for the treatment of spinal muscular atrophy in Spain (RET-AME consensus). *Neurologia*.

[28] Zanoteli E, Araujo APDQC, Becker MM, Fortes CPDD, França MC, Machado-Costa MC (2024). Consensus from the Brazilian Academy of Neurology for the diagnosis, genetic counseling, and use of disease-modifying therapies in 5q spinal muscular atrophy. *Arquivos De Neuro-psiquiatria*.

[29] Nasa P, Jain R, Juneja D (2021). Delphi methodology in healthcare research: How to decide its appropriateness. *World Journal of Methodology*.

[30] Ranganathan P, Caduff C, Frampton CMA (2024). Designing and validating a research questionnaire - Part 2. *Perspectives in Clinical Research*.

[31] Saw SM, Ng TP (2001). The design and assessment of questionnaires in clinical research. *Singapore Medical Journal*.

[32] Prior TW, Professional Practice and Guidelines Committee (2008). Carrier screening for spinal muscular atrophy. *Genetics in Medicine: Official Journal of the American College of Medical Genetics*.

[33] Prior TW (2007). Spinal muscular atrophy diagnostics. *Journal of Child Neurology*.

[34] Oliveira Netto AB, Brusius-Facchin AC, Lemos JF, Pasetto FB, Brasil CS, Trapp FB (2023). Neonatal screening for spinal muscular atrophy: A pilot study in Brazil. *Genetics and Molecular Biology*.

[35] Vill K, Schwartz O, Blaschek A, Gläser D, Nennstiel U, Wirth B (2021). Newborn screening for spinal muscular atrophy in Germany: clinical results after 2 years. *Orphanet Journal of Rare Diseases*.

[36] Cances C, Vlodavets D, Comi GP, Masson R, Mazurkiewicz-Bełdzińska M, Saito K (2022). Natural history of Type 1 spinal muscular atrophy: a retrospective, global, multicenter study. *Orphanet Journal of Rare Diseases*.

[37] Glanzman AM, Mazzone E, Main M, Pelliccioni M, Wood J, Swoboda KJ (2010). The Children’s Hospital of Philadelphia Infant Test of Neuromuscular Disorders (CHOP INTEND): test development and reliability. *Neuromuscular Disorders: NMD*.

[38] Mazzone ES, Mayhew A, Montes J, Ramsey D, Fanelli L, Young SD (2017). Revised upper limb module for spinal muscular atrophy: Development of a new module. *Muscle & Nerve*.

[39] Montes J, McDermott MP, Martens WB, Dunaway S, Glanzman AM, Riley S (2010). Six-Minute Walk Test demonstrates motor fatigue in spinal muscular atrophy. *Neurology*.

[40] Krosschell KJ, Scott CB, Maczulski JA, Lewelt AJ, Reyna SP, Swoboda KJ (2011). Reliability of the Modified Hammersmith Functional Motor Scale in young children with spinal muscular atrophy. *Muscle & Nerve*.

[41] Goedeker NL, Rogers A, Fisher M, Arya K, Brandsema JF, Farah H (2024). Outcomes of early-treated infants with spinal muscular atrophy: A multicenter, retrospective cohort study. *Muscle & Nerve*.

[42] Darras BT, Chiriboga CA, Iannaccone ST, Swoboda KJ, Montes J, Mignon L (2019). Nusinersen in later-onset spinal muscular atrophy: Long-term results from the phase 1/2 studies. *Neurology*.

[43] O’Hagen JM, Glanzman AM, McDermott MP, Ryan PA, Flickinger J, Quigley J (2007). An expanded version of the Hammersmith Functional Motor Scale for SMA II and III patients. *Neuromuscular Disorders: NMD*.

[44] Iannaccone ST, Hynan LS, Morton A, Buchanan R, Limbers CA, Varni JW (2009). The PedsQL in pediatric patients with Spinal Muscular Atrophy: feasibility, reliability, and validity of the Pediatric Quality of Life Inventory Generic Core Scales and Neuromuscular Module. *Neuromuscular Disorders: NMD*.

[45] Zaidman CM, Crockett CD, Wedge E, Tabatabai G, Goedeker N (2024). Newborn Screening for Spinal Muscular Atrophy: Variations in Practice and Early Management of Infants with Spinal Muscular Atrophy in the United States. *International Journal of Neonatal Screening*.

[46] Cooper K, Nalbant G, Sutton A, Harnan S, Thokala P, Chilcott J (2024). Systematic Review of Newborn Screening Programmes for Spinal Muscular Atrophy. *International Journal of Neonatal Screening*.

[47] Glascock J, Sampson J, Connolly AM, Darras BT, Day JW, Finkel R (2020). Revised Recommendations for the Treatment of Infants Diagnosed with Spinal Muscular Atrophy Via Newborn Screening Who Have 4 Copies of SMN2. *Journal of Neuromuscular Diseases*.

[48] McMillan HJ, Proud CM, Farrar MA, Alexander IE, Muntoni F, Servais L (2022). Onasemnogene abeparvovec for the treatment of spinal muscular atrophy. *Expert Opinion on Biological Therapy*.

[49] Administration, U.S.F.a.D (2026). FDA approves new high-dose regimen of SPINRAZA (nusinersen) for spinal muscular atrophy. https://www.fda.gov/.

[50] Finkel RS, Crawford TO, Mercuri E, Sumner CJ, Garcia Romero MDM, Day JW (2026). High-dose nusinersen for spinal muscular atrophy: a phase 3 randomized trial. *Nature Medicine*.

[51] Proud CM, Vũ DC, Wilmshurst JM, Sanmaneechai O, Gulati S, Xiong H (2026). Intrathecal onasemnogene abeparvovec in treatment-naive patients with spinal muscular atrophy: a phase 3, randomized controlled trial. *Nature Medicine*.

[52] Administration, U.S.F.a.D (2026). FDA Label Extension – Evrysdi (Risdiplam). https://www.fda.gov/.

[53] Elshahawy R, Elezbawy B, Ashmawy R, Elshahawy R, Mahmoud YS, Korra N (2025). Global Economic Burden of Spinal Muscular Atrophy: A Systematic Literature Review. *Cureus*.

[54] Alfano LN, Iammarino MA, Reash NF, Powers BR, Shannon K, Connolly AM (2021). Validity and Reliability of the Neuromuscular Gross Motor Outcome. *Pediatric Neurology*.

[55] Pera MC, Coratti G, Forcina N, Mazzone ES, Scoto M, Montes J (2017). Content validity and clinical meaningfulness of the HFMSE in spinal muscular atrophy. *BMC Neurology*.

[56] Yeo CJJ, Simmons Z, De Vivo DC, Darras BT (2022). Ethical Perspectives on Treatment Options with Spinal Muscular Atrophy Patients. *Annals of Neurology*.
